# Epithelium‐Inspired, Ultrahigh‐Toughness, Ultralow‐Hysteresis, and Highly Compressible Polymer Hydrogels as Self‐Powered, Visual, and Underwater Strain Sensors

**DOI:** 10.1002/advs.202510444

**Published:** 2025-12-30

**Authors:** Yutang Zhou, Honghao Shu, Yuhuan Yao, Bolin Lu, Wenbin Zhong

**Affiliations:** ^1^ College of Materials Science and Engineering Hunan University Changsha 410082 China

**Keywords:** epithelium‐like structure, polymer hydrogels, strain sensors, ultrahigh toughness and compressibility, ultralow hysteresis

## Abstract

Developing high‐toughness, low‐hysteresis, and highly compressible polymer hydrogels as wearable strain sensors with superior detection ranges, durability, and signal accuracy is still a grand challenge due to contradictory characteristics. Herein, inspired by epithelial tissue, an epithelium‐like structure hydrogel with cell‐like particles (PLTAV) is synthesized from a water‐in‐oil high internal phase emulsion. During loading‐unloading processes, the hydrophilic cell‐like particles can deform reversibly and be cyclically divided into small‐sized particles and aggregated, thereby dissipating more energy. Therefore, the PLTAV hydrogel with high water content (90.4 wt%) has superior stretchability (1368%), ultrahigh toughness (2.64 MJ·m^−3^), ultralow hysteresis (4.7%, ε = 300%), and ultrahigh compressibility (99.9%). Subsequently, choline chloride and sorbitol are introduced into the PLTAV hydrogel (PLTAV‐SC). The as‐prepared PLTAV‐SC hydrogel exhibits improved freezing resistance, enhanced stretchability (2021%) and toughness (6.10 MJ·m^−3^), and retained low hysteresis and ultrahigh compressibility. Benefiting from the cell‐like particles composed of polymers with ionic structural units and the difference in ionic mobilities, these hydrogels can act as self‐powered strain sensors with high sensitivities. Meanwhile, they can also be used as high‐performance visual and resistance‐type strain sensors. Additionally, these sensors can be further constructed as underwater strain sensors for detecting underwater human/animal motion, water flow velocity, and water depth.

## Graphical abstract

Inspired by epithelial tissue, epithelium‐like structure hydrogels are synthesized. The as‐prepared hydrogels exhibit ultrahigh toughness, ultralow hysteresis, and ultrahigh compressibility, which can be utilized as self‐powered and visual strain sensors. Additionally, resistance‐type strain sensors constructed from the as‐prepared hydrogels have excellent sensing performance, which can be further used for underwater strain sensing.

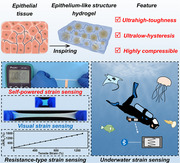

## 1 Introduction

Polymer hydrogel strain sensors (PHSSs) have attracted widespread attention due to their excellent flexibility, high biocompatibility, and tunable conductivity, and have been widely applied in fields such as bionics, robotics, and medicine [1, 2, 3, 4, 5, 6]. However, the further development of conventional PHSSs is limited by their narrow tensile/compressive stress detection ranges, insufficient durability, low linearity, and slow response/recovery [7]. Optimizing only the toughness (related to tensile strength and tensile strain at break), hysteresis, or compressibility of hydrogels can individually enhance one or two of these sensing properties [8, 9, 10]. Thus, it is still a challenge to improve the comprehensive sensing properties of PHSSs based on the high‐toughness, low‐hysteresis, and highly compressible hydrogels prepared by addressing the inherent trade‐offs among mechanical properties [11]. Furthermore, to enable more accurate stress detection, the development of PHSSs with multiple sensing modes is also imperative [12].

To achieve the high toughness of hydrogels, many efforts have been made to improve their tensile strength and tensile strain at break [6, 13, 14, 15, 16, 17, 18, 19, 20]. The main strategies are as follows: 1) constructing sacrificial bonds (e.g., noncovalent interactions and reversible covalent bonds) to dissipate fracture energy and enhance the tensile strain at break [6, 13, 14, 15]; 2) introducing stress‐absorbing components (e.g., crystal domains, micro/nano particles, hydrophobic phases, interpenetrating polymer networks, topological entanglements) via the Hofmeister effect, phase separation, and so on, to improve load transfer and enhance the tensile strength [16, 17, 18, 19, 20]. Notably, abundant sacrificial bonds exist in some stress‐absorbing components, such as *π–π* interactions in hydrophobic phases, which enable the hydrogels to exhibit synergistic toughening through the combined application of these strategies [21]. However, after the deformation of hydrogels, the slow recovery rate of sacrificial bonds and the interchain friction cause significant energy loss and hysteresis [9, 22]. Recently, the solutions have been proposed through improving the aforementioned two strategies. First, sacrificial bonds (e.g., Zn^2+^‐ligand bond, hydrogen bond) with rapid dissociation/association dynamics are adopted [22, 23, 24], which can promote the reconstruction of sacrificial bonds after damage. Second, stress‐absorbing components (e.g., peptides, micelles, porous nanoparticles, microgels, slide rings, stable chain entanglements) with extra chemical/physical crosslinking effects are used [20, 25, 26, 27, 28, 29, 30, 31, 32], which can anchor polymer chains and mitigate interchain friction. Despite significant progress in optimizing toughness and hysteresis recently [28, 33], the compressive strain of the hydrogels is still unsatisfactory (≤90%), possibly due to their high polymer content. The key to enhancing the compressibility lies in improving the deformability of polymer networks by reducing polymer content and crosslinking density, etc [8, 34, 35]. Unfortunately, low polymer content and crosslinking density decrease the tensile strength, compromising the toughness [36]. Based on the above analysis, simultaneously achieving high toughness, low hysteresis, and high compressibility in hydrogels remains an ongoing challenge. As a result, PHSSs with wide tensile/compressive stress detection ranges, excellent durability, high linearity, and fast response/recovery are rarely reported.

Additionally, the application of conventional PHSSs is limited due to the dependence on external power sources, whose integration compromises wearability and escalates operational costs [3, 24]. Recently, the method of generating voltage in hydrogels through different cationic and anionic mobilities under external force, namely the piezoionic effect, has aroused increasing attention due to its simplicity and effectiveness [3, 37, 38]. The generated voltage can not only power other electronics but also respond to mechanical stress and/or strain [3]. Therefore, even without a power supply, PHSSs can still operate effectively using the strategy. To further improve the voltage output and sensing sensitivity of piezoionic sensors, several approaches have been proposed to increase the difference in mobilities between the cations and anions of introduced inorganic salts [3, 37]. Crown ethers matching Na^+^ can increase the difference in mobilities between Na^+^ and Cl^−^ under external force, thereby increasing the voltage output and sensing sensitivity of a piezoionic sensor [3]. A similar result can be obtained by the introduction of K_3_Fe(CN)_6_ and K_4_Fe(CN)_6_ into a piezoionic sensor because the mobilities of Fe(CN)_6_^3−^ and Fe(CN)_6_^4−^ are much lower than that of K^+^ [37]. However, the detection upper limits of these piezoionic sensors for pressure are lower than 1 MPa. Besides, tensile ionic sensors based on hydrogels and the impact of hysteresis on sensing performance are frequently overlooked. Furthermore, although the ions of polyelectrolytes may be easier to separate than those of inorganic salts due to the limited mobility of anionic/cationic polymer segments, it is rarely reported that hydrogels constructed with ionic polymer segments are used as piezo/tensile ionic sensors for generating voltage [38]. Moreover, the effect of phase structures on the voltage output still needs to be further discussed. There is great significance in developing highly compressible hydrogels with ionic polymer segments and special phase structures to construct high‐performance piezo/tensile ionic sensors.

The human skin, as a sophisticated and soft sensory organ, can perceive diverse external stimuli such as strain, pressure, and vibration [3, 39]. Through delving into its intrinsic structure, it is found that cells in epithelial tissue play a vital role in providing mechanical strength and mediating bioelectrical signal transmission [40, 41]. Specifically, keratin filament networks in epithelial cells can enhance the resistance of epithelium to mechanical stress, thereby improving the mechanical strength of the skin [40]. When epithelial cells are mechanically stimulated, cation channels of the cell membranes open, triggering membrane depolarization [41]. Through cell junctions, generated bioelectrical signals can be transmitted to the nervous system. Additionally, epithelial cells possess excellent deformability and superelasticity behavior [42]. Nevertheless, to our knowledge, epithelium‐like structure hydrogels with ultrahigh toughness, ultralow hysteresis, ultrahigh compressibility, and enhanced voltage output have not been reported. Inspired by epithelial tissue, herein, a novel epithelium‐like structure hydrogel (PLTAV) is designed and synthesized. In this hydrogel, hydrophilic cell‐like microgel particles can link hydrophobic polymer segments and decrease interchain friction, thereby reducing the hysteresis of the hydrogel; simultaneously, the microgel particles can endure considerable mechanical stress and strain by self‐deformation, thereby enhancing the toughness and compressibility of the hydrogel. Consequently, the as‐prepared PLTAV hydrogel is expected to have ultrahigh toughness, ultralow hysteresis, and ultrahigh compressibility simultaneously. To expand the applicability of this hydrogel, sorbitol and choline chloride (ChCl) are introduced. Compared to the PLTAV hydrogel, the as‐prepared PLTAV‐SC hydrogel has increased toughness, improved freezing resistance, and retained low hysteresis and high compressibility. By imitating the ion‐regulated sensing processes of epithelial cells, these hydrogels can harvest energy and be constructed as self‐powered strain sensors. Moreover, an aggregation‐induced emission monomer is used as a structural unit, which endows these hydrogels with mechanochromic functions. Thus, the as‐prepared hydrogels can serve as visual strain sensors simultaneously. Furthermore, they can also be utilized as high‐performance resistance‐type strain sensors, which can be further encapsulated by hydrophobic coatings for underwater applications. The presented work offers innovative insights into simultaneously optimizing the toughness, hysteresis, and compressibility of hydrogels and takes a further step in developing high‐performance and multi‐mode strain sensors.

## 2 Results and Discussion

### 2.1 Preparation of PLTAV Hydrogel

**FIGURE 1:**
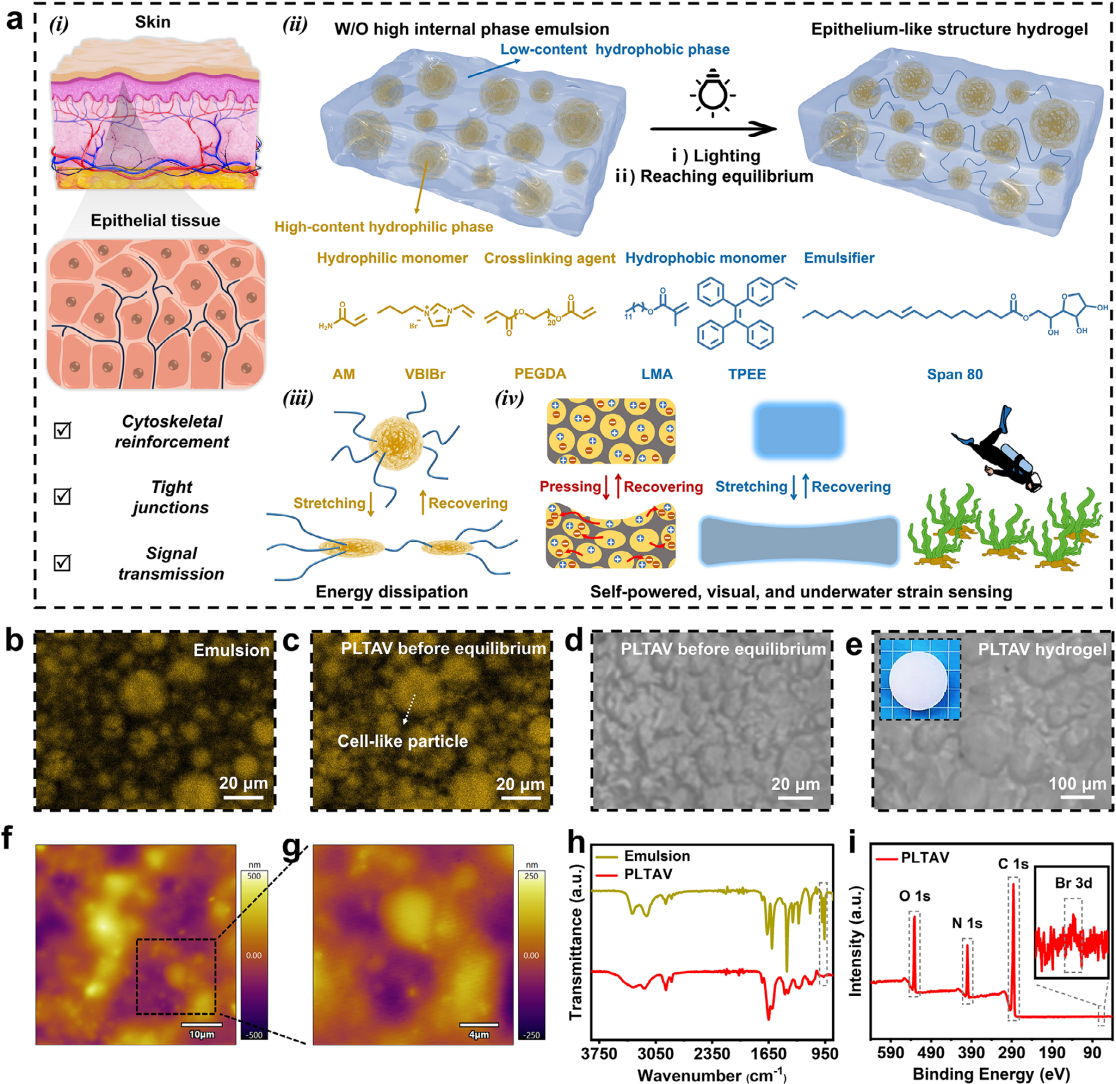
Preparation process and characterization of epithelium‐like structure hydrogel (PLTAV). (a) Schematic illustrations of epithelial tissue, hydrogel preparation process, mechanical energy dissipation pathway, triple sensing modes (self‐powered, visual, underwater). In situ CLSM images of (b) emulsion and (c) PLTAV before equilibrium (Yellow fluorescence stems from hydrophilic Rhodamine B). OM images of (d) PLTAV before equilibrium and (e) PLTAV hydrogel. AFM height images of PLTAV hydrogel at (f) low magnification and (g) high magnification. (h) FTIR and (i) XPS spectra of emulsion and PLTAV hydrogel.

Inspired by epithelial tissue (Figure 1a (i)), a PLTAV hydrogel with cell‐like particles was prepared from a water‐in‐oil high internal phase emulsion (Figure 1a (ii)). The emulsion consisted of monomers (lauryl methacrylate (LMA), [2‐(4‐vinylphenyl)ethene‐1,1,2‐triyl] tribenzene (TPEE), acrylamide (AM), and 1‐vinyl‐3‐butyl imidazole bromide (VBIBr)), a crosslinking agent (poly(ethylene glycol) diacrylate (PEGDA)), and an emulsifier (Span 80). The unique structural change and energy dissipation mechanism of the hydrogel during deformation (Figure 1a (iii)) are conductive to achieve high toughness, low hysteresis, and high compressibility. Besides, the hydrogel can be used as self‐powered, visual, underwater strain sensors (Figure 1a (iv)). The morphology and structure of the PLTAV hydrogel are investigated by confocal laser scanning microscope (CLSM), optical microscope (OM), atomic force microscope (AFM), Fourier transform infrared spectroscopy (FTIR), and X­ray photoelectron spectroscopy (XPS). In CLSM tests, hydrophilic Rhodamine B is adopted as a fluorescence probe. As shown in in situ CLSM images (Figure 1b), spherical particles are observed in the emulsion. After polymerization, they are turned into cell‐like microgel particles and emit stronger yellow fluorescence (Figure 1c), which demonstrates that the particles are hydrophilic. After reaching swelling equilibrium, the as‐prepared PLTAV hydrogel exhibits a high water content of 90.4 wt.% and an increased particle size as seen in OM images (Figure 1d,e). With the increase of drying time, the particle size continuously decreases (Figure S1), which further demonstrates the hydrophilicity of the particles. AFM height images of the PLTAV hydrogel at low magnification also confirm the formation of an epithelium‐like structure (Figure 1f). At high magnification, “epithelial cells” of varying sizes are clearly observed (Figure 1g). Compared to the FTIR spectrum of the emulsion (Figure 1h), the characteristic peak of = C─H (985 cm^−1^) cannot be observed in that of the PTAV hydrogel, which indicates that the monomers are successfully copolymerized. From the XPS spectra (Figure 1i), the PLTAV hydrogel is formed by C, N, O, and Br elements. To highlight the advantages of the PLTAV hydrogel, a hydrogel prepared in N,N‐Dimethylformamide and exchanged solvents with water (SE) and a hydrogel without hydrophobic structural units of LMA and TPEE (PAV) were synthesized for comparison (Figure S2). There are no cell‐like particles in these hydrogels in the OM images. Additionally, the ionic conductivity of the PLTAV hydrogel is higher than that of the SE and PAV hydrogels (Figure S3). The result may be caused by the particles of the PLTAV hydrogel, which can form conductive channels and promote ion transport under an applied electric field. The FTIR spectrum of the SE hydrogel closely resembles that of the PLTAV hydrogel due to the consistent components (Figure S4). However, the characteristic peaks of ‐C═O (1729 cm^−1^) and C─H (2852 cm^−1^) of LMA cannot be discovered in the FTIR spectrum of the PAV hydrogel. The result is attributed to the lack of hydrophobic components.

### 2.2 Mechanical Properties of PLTAV Hydrogel

**FIGURE 2:**
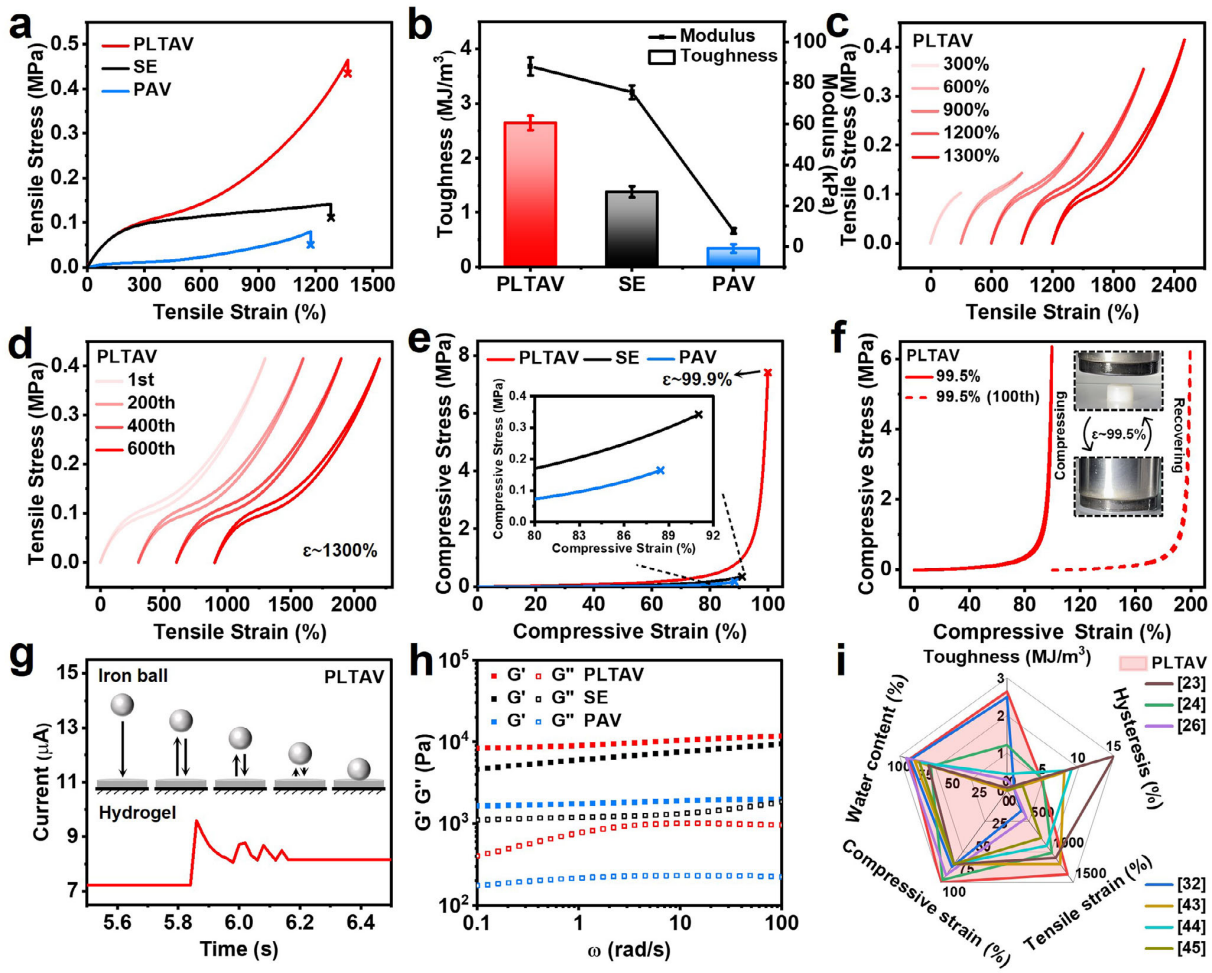
Mechanical properties of PLTAV hydrogel. (a) Tensile stress‐strain curves of PLTAV, SE, and PAV hydrogels. (b) Toughness and modulus of PLTAV, SE, and PAV hydrogels. (c) Tensile stress‐strain curves of PLTAV hydrogel during loading‐unloading processes at different strains. (d) Tensile stress‐strain curves of PLTAV hydrogel during successive loading‐unloading processes at a 1300% strain. (e) Compressive stress‐strain curves of PLTAV, SE, and PAV hydrogels. (f) Compressive stress‐strain curves of PLTAV hydrogel during successive loading–unloading processes at a 99.5% strain. (g) Real‐time current changes of PLTAV hydrogel during the impact of a falling iron ball (Weight: 5 g; Initial height: 15 cm). (h) Gʹ and Gʺ of PLTAV, SE, and PAV hydrogels by an angular frequency of sweep test at a 1% strain. (i) Comparison of water content, toughness, hysteresis, tensile strain, and compressive strain of PLTAV hydrogel with previously reported hydrogels.

The mechanical properties of PLTAV, SE, and PAV hydrogels are characterized and analyzed, as described in Figure 2. From Figure 2a,b, the tensile strength, tensile strain at break, toughness, and modulus of the PLTAV hydrogel (0.46 MPa, 1368%, 2.64 MJ·m^−3^, 88.13 kPa) exceed those of the SE (0.14 MPa, 1279%, 1.38 MJ·m^−3^, 75.44 kPa) and PAV (0.05 MPa, 1173%, 0.34 MJ·m^−3^, 7.71 kPa) hydrogels. Furthermore, as shown in Figure S5a–c, the as‐prepared PLTAV hydrogel exhibits superior deformability, cutting resistance, and weight‐bearing capacity. Subsequently, the tensile stress‐strain curves of the PLTAV hydrogel during loading‐unloading processes at different strains are measured. As presented in Figure 2c, hysteresis loops cannot be clearly observed. As the strain increases from 300% to 1300%, the hysteresis ratio rises gradually from 4.7 to 7.5% (Figure S6a). After 600 cycles at a 1300% strain, the as‐prepared PLTAV hydrogel still retains ultralow hysteresis (Figure 2d; Figure S6b). In contrast to the PLTAV hydrogel, the SE and PAV hydrogels display obvious hysteresis loops at a tensile strain of 900% (Figure S7a). Their hysteresis ratios are 52.5% and 16.5%, respectively, much higher than the 6.3% of the PLTAV hydrogel (Figure S7b). Besides tensile properties, the compressive behavior of the hydrogels is also investigated. As shown in Figure 2e, the compressive strain at break of the PLTAV hydrogel reaches 99.9%, surpassing those of the SE (91.0%) and PAV (88.4%) hydrogels. The corresponding compressive strength is up to 7.4 MPa. The compressive stress‐strain curves of the PLTAV hydrogel during loading‐unloading processes at different strains also indicate the low hysteresis of the hydrogel (Figure S8a,b). Even at a 99.5% strain, the PLTAV hydrogel retains excellent elasticity and durability (Figure 2f). As denoted in Figure S9a,b, the hysteresis ratios of SE (52.1%) and PAV (20.9%) hydrogels also exceed the 9.7% of the PLTAV hydrogel at an 80% compressive strain. Furthermore, the elasticities of hydrogels are analyzed by observing the processes of an iron ball falling in real time. Figure 2g, Figure S10a,b reveal that the rebound count of the PLTAV hydrogel is higher than that of the SE and PAV hydrogels, which implies its lower hysteresis and higher elasticity. Based on the rheological study (Figure 2h), the storage modulus (Gʹ) of the PLTAV hydrogel is significantly higher than its loss modulus (Gʺ) and the Gʹ of other hydrogels, which further supports its high elasticity. In contrast with previously reported low‐hysteresis hydrogels developed by other design strategies (Figure 2i) [23, 24, 26, 32, 43, 44, 45], the as‐prepared PLTAV hydrogel inspired by epithelial tissue demonstrates significant advantages in water content, toughness, hysteresis, stretchability, and compressibility (more details are provided in Tables S3 and S4).

### 2.3 Effect of Cell‐Like Particles on Mechanical Properties

**FIGURE 3:**
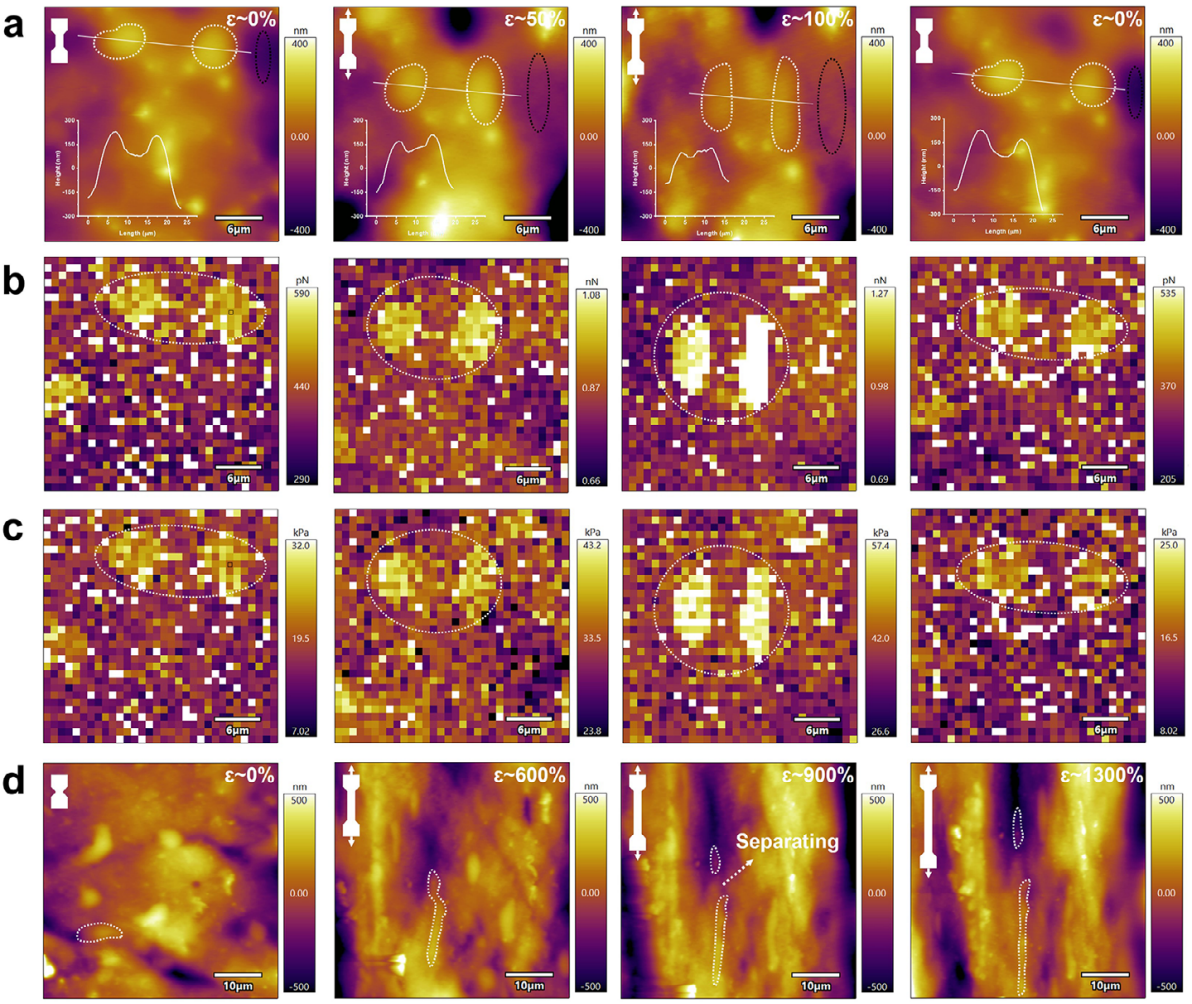
Morphological changes in PLTAV hydrogel during stretching and recovery. In situ AFM (a) height, (b) adhesion, and (c) modulus images of PLTAV hydrogel during stretching and recovery. (d) In situ AFM height images of PLTAV hydrogel with the increase of tensile strain from 0% to 1300%.

To understand the effect of cell‐like particles on mechanical properties, AFM and OM are employed to observe morphological changes in the PLTAV hydrogel during stretching and recovery. As shown in AFM height images (Figure 3a; Figure S11), bright and dark regions are observed, corresponding to hydrophilic cell‐like particles and hydrophobic microdomains with low polymer content, respectively. Owing to Poisson's effect, the particles elongate and narrow during stretching, and their height also decreases. Meanwhile, the dark regions progressively brighten, and the internal microstructure is gradually exposed. When the external force is removed, these particles fully revert to their original morphology, which indicates their exceptional deformability. The reversible behavior is further confirmed by OM images (Figure S12). As presented in AFM adhesion images (Figure 3b), the adhesion force between cell‐like particles and the probe increases continuously as strain increases from 0% to 100%. The trend is attributed to more functional groups on the particle surface, resulting from the extension of initially curled hydrophilic polymer segments in the particles. When strain is reduced to 0%, the adhesion force declines, which indicates the shrinkage of the extended polymer segments and proves the remarkable recoverability of the particles. Since the modulus of hydrophilic particles is higher than that of the hydrophobic microdomain (Figure 3c), applied loads are predominantly distributed over the cell‐like particles (similar to cytoskeletal reinforcement) [46]. Thus, strain‐reinforcing behavior in these particles can be found in AFM modulus images; that is, stretching elevates the modulus of the particles, which suggests that the particles can act as stress‐absorbing components to improve load transfer and prevent crack propagation. Remarkably, the modulus recovers after load removal, which implies low energy loss during particle deformation. When the tensile strain reaches 900%, it can be observed in Figure 3d that the separation of the cell‐like particle into two distinct parts, which indicates that a large cell‐like particle may be assembled from multiple small‐sized particles linked by polymer chains (similar to epithelial tight junctions).This separation process can dissipate more fracture energy, thereby enabling the hydrogel to withstand larger deformation [33, 47]. Notably, when the strain is further increased to 1300%, the separated particles remain deformable. Furthermore, after multiple cycles at a 1300% strain (Figure 2d), the mechanical performance of PLTAV hydrogel has not changed significantly, which implies the excellent reversible deformability of cell‐like particles. Figures S13 and S14 demonstrate that the cell‐like particles align orderly along the stretching direction at a high tensile strain, which can effectively reinforce the polymer network [48].

Based on the above results and discussion, the reasons for the superior mechanical properties of the PLTAV hydrogel are as follows. First, the cell‐like microgel particles with outstanding reversible deformability can enable the PLTAV hydrogel to withstand higher stress and strain while maintaining low energy loss and obtain comprehensive improvement of mechanical properties. Second, in contrast with the PAV hydrogel, the PLTAV and SE hydrogels are composed of hydrophobic microdomains, which can also serve as stress‐absorbing components to endow the hydrogels with high strength and modulus [18]. Besides, the *π–π* interaction in the hydrophobic microdomain can dissipate fracture energy, thereby increasing the strain at break and toughness of the hydrogels [21]. Third, compared to the SE hydrogel, the hydrophobic microdomain of the PLTAV hydrogel is formed in situ and connected by hydrophilic microgel particles. This decreases unstable chain entanglements in the hydrogel, which minimizes interchain friction and energy loss, thereby reducing the hysteresis of the hydrogel [20, 30].

### 2.4 Freezing Resistance and Mechanical Performance of PLTAV‐SC Hydrogel

**FIGURE 4:**
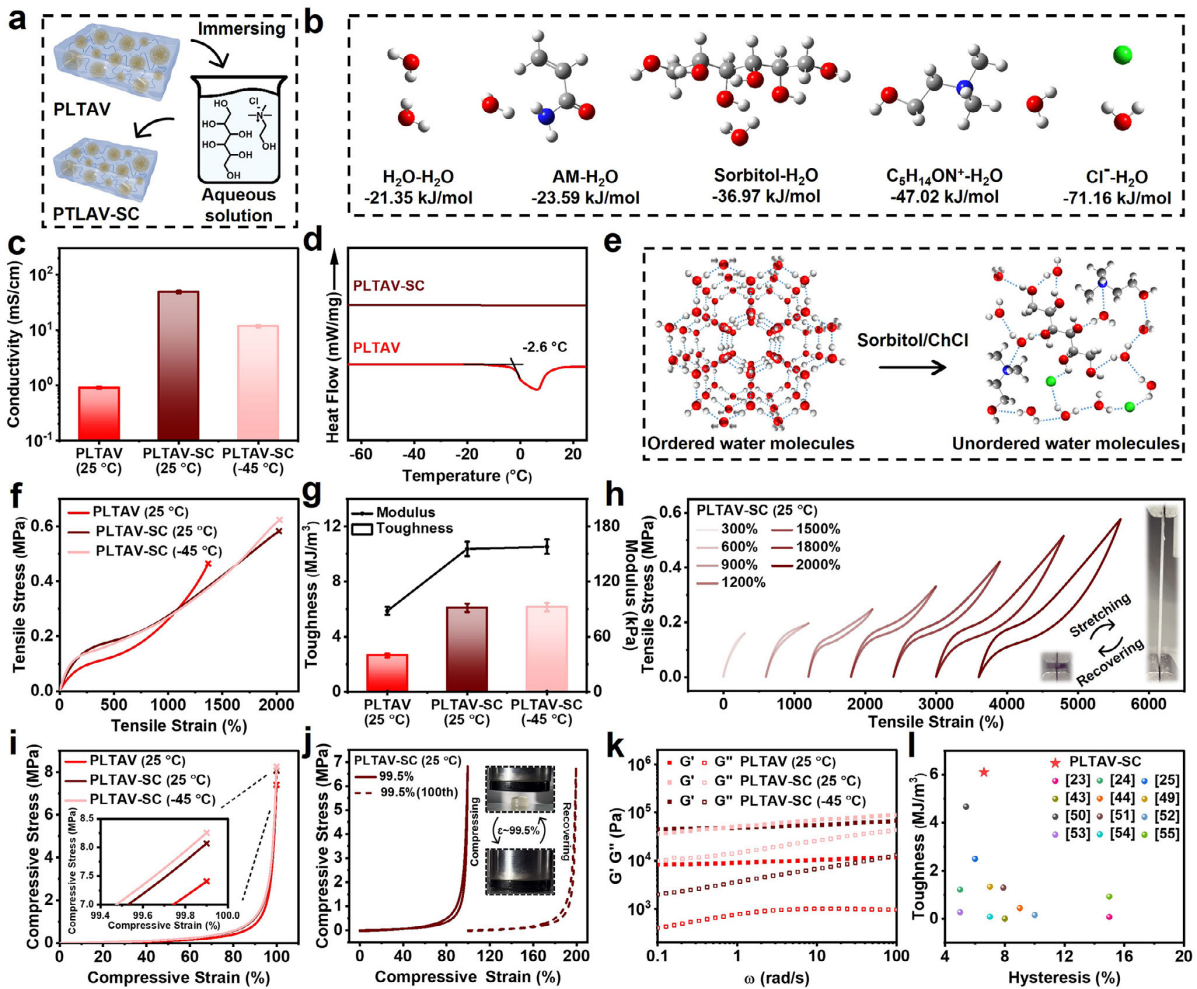
Preparation, anti‐freezing ability, and mechanical performance of PLTAV‐SC hydrogel. (a) Preparation process of PLTAV‐SC hydrogel. (b) Binding energy of H_2_O with different components. (c) Ionic conductivity of PLTAV and PLTAV‐SC hydrogels at different temperatures. (d) DSC results of PLTAV and PLTAV‐SC hydrogels. (e) Effect of ChCl and sorbitol on water molecules at −45°C. (f) Tensile stress‐strain curves of PLTAV and PLTAV‐SC hydrogels at different temperatures. (g) Toughness and modulus of PLTAV and PLTAV‐SC hydrogels at different temperatures. (h) Tensile stress‐strain curves of PLTAV‐SC hydrogel during loading‐unloading processes at different strains. (i) Compressive stress‐strain curves of PLTAV and PLTAV‐SC hydrogels at different temperatures. (j) Compressive stress‐strain curves of PLTAV‐SC hydrogel during successive loading‐unloading processes at a 99.5% strain. (k) Gʹ and Gʺ of PLTAV and PLTAV‐SC hydrogels by an angular frequency of sweep test at a 1% strain at different temperatures. (l) Comparison of toughness and hysteresis of PLTAV‐SC hydrogel with previously reported gels.

To realize the all‐weather application of the PLTAV hydrogel, its freezing resistance needs to be considered. Thus, an anti‐freezing PLTAV‐SC hydrogel was developed by immersing the PLTAV hydrogel in a mixed aqueous solution of sorbitol and choline chloride (ChCl) (Figure 4a). As shown in Figures S15 and S16, the ratio and concentration of sorbitol and ChCl were optimized. Based on density functional theory (DFT) calculations (Figure 4b), the binding energy of H_2_O with sorbitol and ChCl is lower than that of H_2_O with H_2_O, which means that sorbitol and ChCl can inhibit ice crystal formation by suppressing interactions among water molecules at low temperatures (the hydration effect). When the ambient temperature drops from 25°C to −45°C, the PLTAV‐SC hydrogel remains flexible, whereas the PLTAV hydrogel becomes hard (Figure S17). As revealed in Figure 4c, the ionic conductivities of the PLTAV and PLTAV‐SC hydrogels are 0.9 and 49.0 mS·cm^−1^, respectively. At −45°C, the ionic conductivity of the PLTAV‐SC hydrogel retains 11.7 mS·cm^−1^. By comparing differential scanning calorimetry (DSC) curves of the PLTAV and PLTAV‐SC hydrogels (Figure 4d), it can be found that the PLTAV‐SC hydrogel has no peaks in the range of −65°C–25°C, but the PLTAV hydrogel exhibits a prominent peak at −2.6°C. These results elucidate that the introduction of sorbitol and ChCl significantly improves the freezing resistance of the PLTAV hydrogel. That is, their hydration effect reduces free water content and disrupts the ordered arrangement of water molecules (Figure 4e). Meanwhile, ChCl, as an organic salt, also increases the ionic conductivity of the hydrogel. For the same reason, the PLTAV‐SC hydrogel will possess superior moisturizing ability. Besides, the binding energy of H_2_O with sorbitol and ChCl is also lower than that of H_2_O with AM (Figure 4b). The result implies that the interaction of sorbitol and ChCl with H_2_O is stronger than that of AM with H_2_O, inducing the shrinkage and aggregation of hydrophilic polymer segments (the salting‐out effect). As displayed in FTIR spectra (Figure S18), the characteristic peaks of N‐H and C═O of the PLTAV hydrogel have shifted after introducing sorbitol and ChCl, which indicates the formation of more hydrogen bonds. Compared to the XPS spectrum of the PLTAV hydrogel (Figure S19), that of the PLTAV‐SC hydrogel has an extra peak corresponding to Cl 2p, which suggests the successful incorporation of the mixed solution of ChCl and sorbitol. From XPS C 1s, N 1s, and O 1s spectra (Figure S20), the C═O and C─N peaks of the PLTAV hydrogel also have changed after the introduction of sorbitol and ChCl, which further demonstrates the increase of hydrogen bonds. The in situ AFM images of the PLTAV and PLTAV‐SC hydrogels are presented in Figure S21. Compared to cell‐like particles in the PLTAV hydrogel, the particles in the PLTAV‐SC hydrogel have a smaller size and higher adhesion force and modulus. Additionally, the transparency of the PLTAV‐SC hydrogel is slightly higher than that of the PLTAV hydrogel (Figure S22).

Subsequently, the mechanical properties of the PLTAV‐SC hydrogel are evaluated at different temperatures. As presented in Figure 4f, the tensile strength and tensile strain at break (0.58 MPa and 2021%) of the PLTAV‐SC hydrogel are higher than those of the PLTAV hydrogel. Its toughness and modulus (6.10 MJ·m^−3^ and 155.52 kPa) are about twice those of the PLTAV hydrogel (Figure 4g). The result is ascribed to the salting‐out effect [16]. Specifically, the effect leads to the aggregation of hydrophilic polymer segments, and formed aggregates can also act as stress‐absorbing components to enhance the strength and modulus of the hydrogel. Simultaneously, the salting‐out effect may reduce the number of scattered polymer chains and increase polymer chain entanglements, which can dissipate more fracture energy and improve the strain at break and toughness of the hydrogel [28]. Notably, at −45°C, there is no significant change in these properties of the PLTAV‐SC hydrogel owing to its outstanding freezing resistance. Hysteresis loops of the PLTAV‐SC hydrogel at different tensile strains are illustrated in Figure 4h. Corresponding hysteresis ratios are calculated and recorded in Figure S23. Within the strain range of 300%–1200%, the as‐prepared PLTAV‐SC hydrogel possesses low hysteresis (6.6%–10.9%), slightly higher than that of the PLTAV hydrogel. The result is attributed to the interactions among sorbitol, ChCl, and the polymer chains (Figure S24), increasing the viscosity of the hydrogel. When the strain reaches 1800% and 2000%, the hysteresis ratio increases to 19.0% and 25.6%, respectively. This phenomenon may be caused by the tight stacking of polymer chains at large deformations, which leads to a significant increase in the interchain friction. At ‐45°C, the hysteresis of the PLTAV‐SC hydrogel is relatively high (Figure S25), which is ascribed to the increase in viscosity. The compressive stress‐strain curves of the PLTAV‐SC hydrogel at different temperatures are shown in Figure 4i. At 25°C and −45°C, the compressive strength at break of the PLTAV‐SC hydrogel is up to 8.0 and 8.2 MPa, respectively, higher than that of the PLTAV hydrogel. Meanwhile, its compressive strain at break remains as high as 99.9%. As seen in Figure S26a,b, the as‐prepared PLTAV‐SC hydrogel exhibits low hysteresis at different compressive strains. Even at a 99.5% strain, the PLTAV‐SC hydrogel maintains exceptional elasticity and durability (Figure 4j). The hysteresis of the PLTAV‐SC hydrogel is slightly higher than that of the PLTAV hydrogel at an 80% compressive strain at 25°C and −45°C (Figure S27). The dynamic viscoelasticity of the PLTAV‐SC hydrogel is evaluated at different temperatures. As shown in Figure 4k, the Gʹ of the PLTAV‐SC hydrogel is always higher than its Gʺ in the range of 0.1–100 rad·s^−1^ at 25°C and −45°C, which further confirms its superior elasticity and freezing resistance. Remarkably, as illustrated in Figure 4l, the toughness and hysteresis of the as‐prepared PLTAV‐SC hydrogel are superior to those of previously reported low‐hysteresis gels (more details are recorded in Table S3) [23, 24, 25, 43, 44, 49, 50, 51, 52, 53, 54, 55].

### 2.5 PLTAV and PLTAV‐SC Hydrogels as Self‐Powered Strain Sensors

**FIGURE 5:**
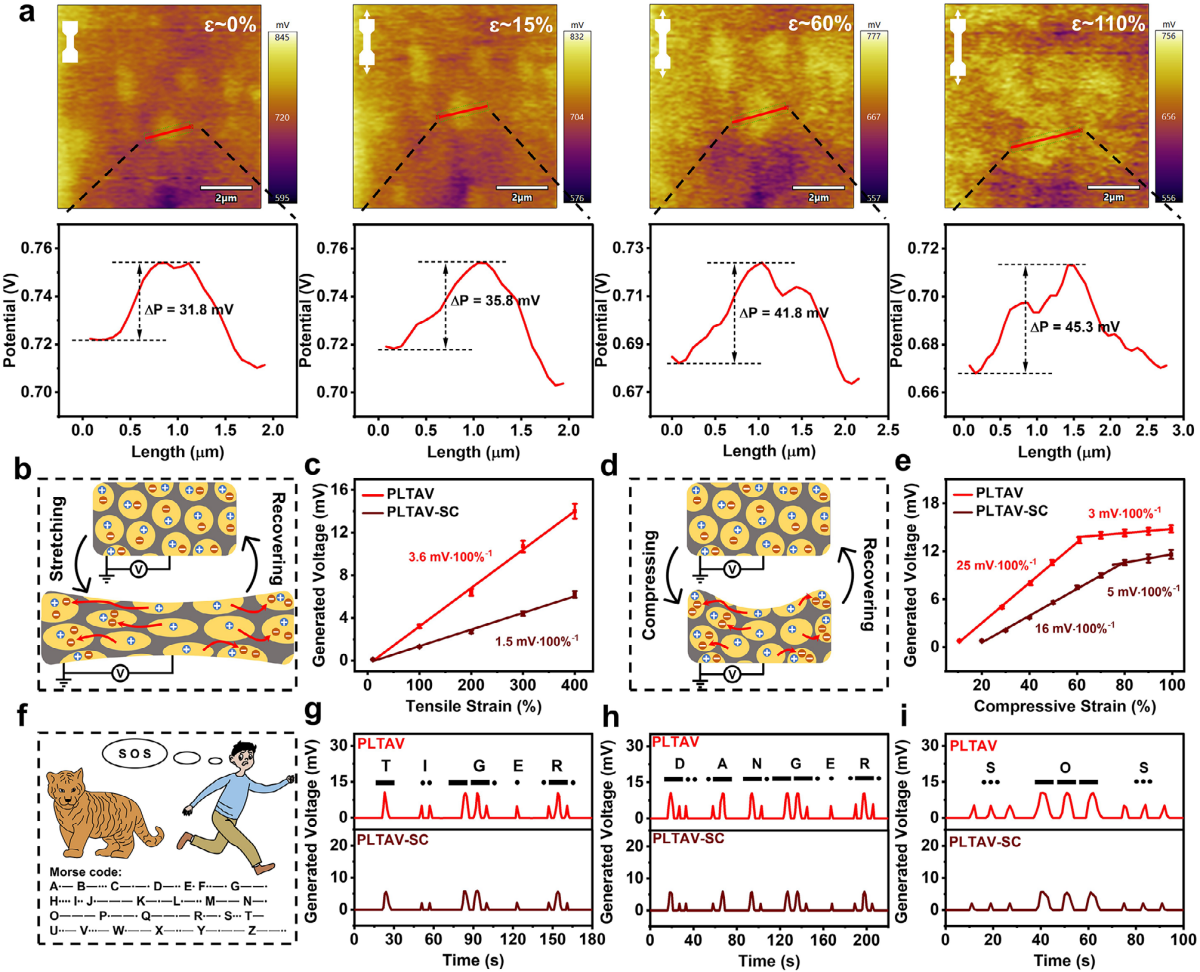
Self‐powered sensing properties and potential applications of PLTAV and PLTAV‐SC hydrogel strain sensors. (a) In situ KPFM surface potential images of PLTAV hydrogel during stretching. (b) Mechanism diagram of PLTAV hydrogel for generating voltage through stretching. (c) Generated voltages of PLTAV and PLTAV‐SC sensors with different tensile strains. (d) Mechanism diagram of PLTAV hydrogel for generating voltage through compression. (e) Generated voltages of PLTAV and PLTAV‐SC sensors with different compressive strains. (f) PLTAV and PLTAV‐SC sensors for information communication. Pressing PLTAV and PLTAV‐SC sensors to transmit different information, including (g) “TIGER”, (h) “DANGER”, and (i) “SOS”.

The Kelvin probe force microscopy (KPFM) surface potential images of the PLTAV hydrogel at different tensile strains are shown in Figure 5a. To our knowledge, this is the first time KPFM has been employed to observe potential changes in the hydrogel during stretching. Bright and dark regions represent the cell‐like particle with high potential and the hydrophobic microdomain with low potential, respectively, owing to the presence of cationic polymer segments and free anions. With the increase of tensile strain, the bright regions gradually increase due to the extension of the particles. Furthermore, the potential difference between the cell‐like particle and the hydrophobic microdomain also increases. The possible mechanism is illustrated in Figure 5b. When a tensile strain is applied to the PLTAV hydrogel, the particles are squeezed, and Br^−^ migrates along the stretching direction. Due to the restricted mobility of cationic polymer chains, the potential of the cell‐like particle increases. Besides, the migration of Br^−^ also leads to the generation of voltage between the middle and side of the PLTAV hydrogel, which enables the PLTAV hydrogel to be a promising candidate for self‐powered strain sensors. As exhibited in Figure 5c, the PLTAV hydrogel is constructed as the first reported tensile ionic hydrogel strain sensor, and its sensitivity to tensile strain is 3.6 mV·100%^−1^. Moreover, the potential difference between the cell‐like particle and the hydrophobic microdomain also increases during the stretching of the PLTAV‐SC hydrogel (Figure S28). However, the sensitivity of the PLTAV‐SC sensor is 1.5 mV·100%^−1^, lower than that of the PLTAV sensor (Figure 5c). The reason for this result is that more C_5_H_14_ON^+^ and Cl^−^ shield the influence of cationic polymer segments and Br^−^ on the generation of voltage, resulting in the reduction of the difference in cationic and anionic mobilities. Besides stretching, compression can also enable these hydrogels to generate voltage (Figure S29). The underlying mechanism is indicated in Figure 5d and is similar to the mechanism of generating voltage through stretching the hydrogels. As displayed in Figure 5e, the maximum sensitivity of the PLTAV sensor to compressive strain is 25 mV·100%^−1^, higher than that of the PLTAV‐SC sensor (16 mV·100%^−1^). Notably, the PLTAV and PLTAV‐SC sensors have ultrahigh detection upper limits for compressive strain (99.5%), corresponding pressures of 6.4 MPa and 6.9 MPa, respectively. At −45°C, the maximum sensitivity of the PLTAV‐SC sensor increases to 40 mV·100%^−1^ due to the increase of the difference in mobilities between C_5_H_14_ON^+^ and Cl^−^ (Figure S30). Moreover, the generated voltage of the PLTAV sensor (8.0 mV) at a 40% compressive strain is larger than that of the SE and PAV sensors (2.7 and 4.6 mV), as exhibited in Figure S31. The result is attributed to the cell‐like particles in the PLTAV sensor, which form conductive channels and facilitate Br^−^ separation, thereby improving ion transport efficiency. For the same reason, the as‐prepared sensors have higher generated voltages compared with crown ether grafted polyvinyl alcohol/sodium chloride (PVA‐CE/NaCl) and polyacrylamide/sodium chloride (PAM/NaCl) sensors [3, 38]. The generated voltages of the PLTAV and PLTAV‐SC sensors show negligible fluctuations at different compressive speeds (Figure S32a) and remain stable over multiple loading‐unloading cycles (Figure S32b), which suggests that the as‐prepared sensors possess excellent reliability and durability. Moreover, the response time of each sensor is nearly identical to the corresponding recovery time, which is possibly related to the low hysteresis of the hydrogels. Interestingly, these self‐powered sensors can also be applied in encrypted communication. As disclosed in Figure 5f, Morse code combines dots and dashes in different orders to represent various letters. In this system, the generated voltages corresponding to minor and large compressive strains are assigned to dots and dashes, respectively. When the personal safety of users is threatened, they can transmit different information by pressing the sensors in sequences according to Morse code, such as “TIGER”, “DANGER”, and “SOS” (Figure 5g–i).

### 2.6 PLTAV and PLTAV‐SC Hydrogels as Visual Strain Sensors

**FIGURE 6:**
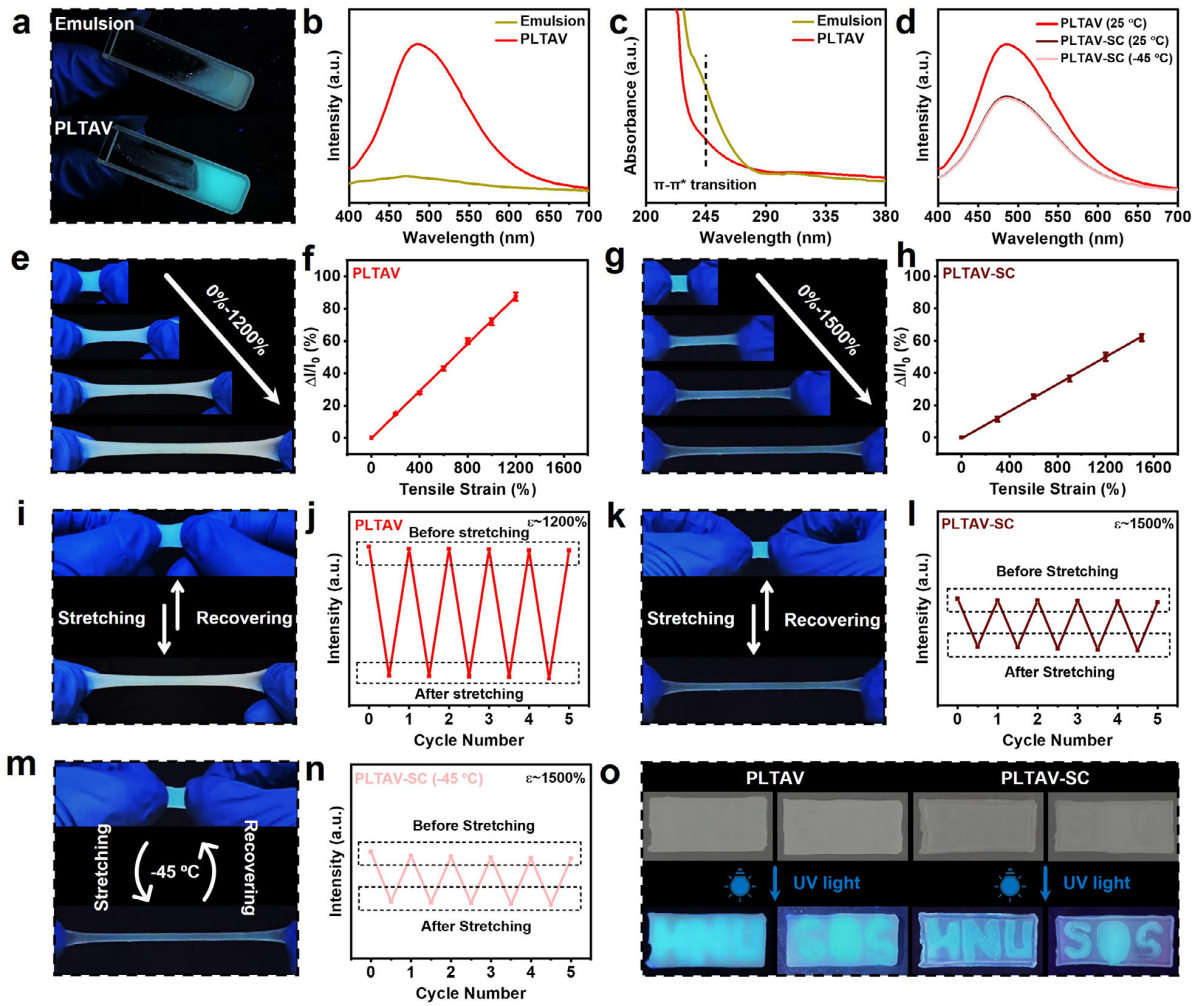
Visual sensing properties and potential applications of PLTAV and PLTAV‐SC hydrogel strain sensors. (a) Digital images of emulsion and PLTAV hydrogel under 365 nm UV light. (b) Fluorescence emission spectra and (c) UV absorption spectra of emulsion and PLTAV hydrogel. (d) Fluorescence emission spectra of PLTAV and PTAV‐SC hydrogels at different temperatures. (e) Digital images of PLTAV hydrogel at different tensile strains under 365 nm UV light. (f) Relative fluorescence intensity variation of PLTAV sensor with tensile strain. (g) Digital images of PLTAV‐SC hydrogel at different tensile strains under 365 nm UV light. (h) Relative fluorescence intensity variation of PLTAV‐SC sensor with tensile strain. (i) Digital images of PLTAV sensor during stretching and recovery under 365 nm UV light. (j) Maximum fluorescence intensity of PLTAV sensor during successive stretching‐recovering processes at a 1200% tensile strain. (k) Digital images of PLTAV‐SC sensor during stretching and recovery under 365 nm UV light. (l) Maximum fluorescence intensity of PLTAV‐SC sensor during successive stretching‐recovering processes at a 1500% tensile strain. (m) Digital images of PLTAV‐SC sensor during stretching and recovering under 365 nm UV light at −45°C. (n) Maximum fluorescence intensity of PLTAV‐SC sensor during successive stretching‐recovering processes at a 1500% tensile strain at −45°C. (o) PLTAV and PLTAV‐SC sensors for visual communication and information encryption.

Recently, visible signals, instead of electrical signals, have been widely used to respond to temperature, deformation, water content, and fatigue damage in hydrogels [39, 48, 56, 57, 58, 59, 60, 61]. Therefore, to possess multiple sensing modes and achieve more accurate sensing, the function of outputting visible signals has been introduced into polymer hydrogel strain sensors (PHSSs) [39, 48, 62, 63]. Due to the incorporation of TPEE, a typical aggregation‐induced emission (AIE) molecule [64], the as‐prepared hydrogels are expected to emit strong fluorescence. As seen in Figure 6a,b, the emulsion (before PLTAV hydrogel formation) can emit weak fluorescence, whereas the PLTAV hydrogel can exhibit strong blue fluorescence. The result can be attributed to the formation of hydrophobic polymer segments, which greatly restrict the rotation of TPEE functional groups and induce the AIE effect. For the same reason, compared to the ultraviolet (UV) absorption spectrum of the emulsion (Figure 6c), that of the PLTAV hydrogel has no distinct absorption band corresponding to the *π–π* ^*^ transition around 245 nm [65]. Additionally, the fluorescence intensity of PLTAV‐SC hydrogel is slightly lower than that of the PLTAV hydrogel due to the increased transmittance (Figure 6d) [66]. At −45°C, the fluorescence intensity of the PLTAV‐SC remains constant, which suggests that low temperatures will not cause the formation of extra TPEE aggregates. As seen in Figure 6e and Figure S33a, as the tensile strain increases, the fluorescence intensity of the PLTAV hydrogel gradually weakens. This phenomenon is attributed to the extension of polymer chains caused by the deformation of cell‐like particles (Figure S34), which disrupts the TPEE aggregates and weakens the AIE effect [67]. Therefore, the as‐prepared PLTAV hydrogel can act as a visual strain sensor. The relative fluorescence intensity variation of the PLTAV visual sensor with different tensile strains can be observed in Figure 6f. Within the strain range of 0%–1200%, the relative fluorescence intensity variation of the PLTAV sensor exhibits excellent linearity. Similar behavior is also observed in the PLTAV‐SC hydrogel (Figure 6g; Figure S33b). Compared to the PLTAV sensor, the PLTAV‐SC sensor possesses relatively lower sensitivity but a wider linear strain range. After the removal of external force, the PLTAV sensor shape recovers, the TPEE reaggregates, and the sensor emits original strong blue fluorescence again (Figure 6i). During repeated stretching‐recovering cycles at a 1200% strain, the variation of maximum fluorescence intensity of the PLTAV sensor remains nearly consistent (Figure 6j). Meanwhile, the PLTAV‐SC sensor can also repeatedly emit fluorescence during loading‐unloading processes (Figure 6k,l). Even at −45°C, the PLTAV‐SC sensor maintains reliable and repeatable visual strain sensing capabilities (Figure 6m,n). Besides constructing visual strain sensors, the as‐prepared hydrogels can enable visual communication. As displayed in Figure 6o, the PLTAV and PLTAV‐SC hydrogels can be prepared as letters with relevant information and embedded on TPEE‐free hydrogels. In emergency scenarios, users can transmit fluorescence‐based signals to request external assistance through these hydrogels. Moreover, under visible light, the letters on these hydrogels cannot be seen. Thus, the as‐prepared hydrogels can also be applied in information encryption.

### 2.7 PLTAV and PLTAV‐SC Hydrogels as Resistance‐Type and Underwater Strain Sensors

**FIGURE 7:**
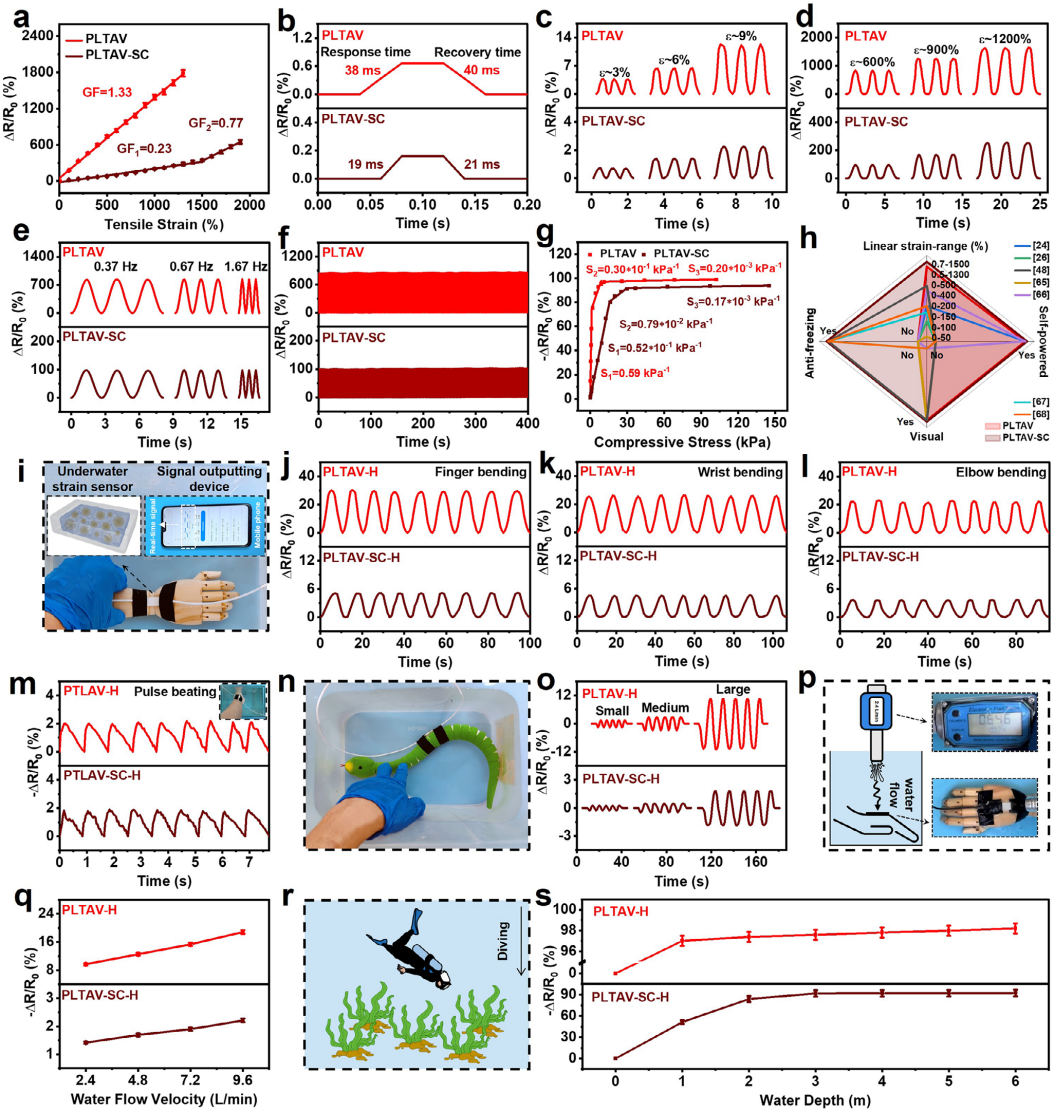
Resistance‐type sensing properties and potential underwater applications of PLTAV and PLTAV‐SC hydrogel strain sensors. (a) Relative resistance changes of PLTAV and PLTAV‐SC sensors with tensile strain. (b) Response and recovery times of PLTAV and PLTAV‐SC sensors during loading and unloading. Relative resistance changes of PLTAV and PLTAV‐SC sensors at (c) minor tensile strains (3%, 6%, and 9%) and (d) large tensile strains (600%, 900%, and 1200%). (e) Relative resistance changes of PLTAV and PLTAV‐SC sensors at a 600% tensile strain under different frequencies. (f) Relative resistance changes of PLTAV and PLTAV‐SC sensors during loading‐unloading cycles at a 600% tensile strain. (g) Relative resistance changes of PLTAV and PLTAV‐SC sensors with compressive stress. (h) Comparison of linear strain range, self‐powered strain sensing, visual strain sensing, and anti‐freezing abilities of PLTAV and PLTAV‐SC strain sensors with previously reported strain sensors. (i) PLTAV‐H and PLTAV‐SC‐H sensors for underwater human motion detection. Variation of real‐time relative resistance of PLTAV‐H and PLTAV‐SC‐H sensors with (j) finger, (k) wrist, and (l) elbow bending. (m) Variation of real‐time relative resistance of PLTAV‐H and PLTAV‐SC‐H sensors with wrist pulse beating. (n) PLTAV‐H and PLTAV‐SC‐H sensors for underwater animal motion detection. (o) Variation of real‐time relative resistance of PLTAV‐H and PLTAV‐SC‐H sensors with water snake swinging. (p) PLTAV‐H and PLTAV‐SC‐H sensors for water flow velocity detection. (q) Variation of relative resistance of PLTAV‐H and PLTAV‐SC‐H sensors with water flow velocity. (r) PLTAV‐H and PLTAV‐SC‐H sensors for water depth detection. (s) Variation of relative resistance of PLTAV‐H and PLTAV‐SC‐H sensors with water depth.

In addition to serving as self‐powered and visual strain sensors, the PLTAV and PLTAV‐SC hydrogels can also act as resistance‐type strain sensors. As illustrated in Figure 7a, the relative resistance of the PLTAV and PLTAV‐SC sensors increases with increasing tensile strain. The PLTAV sensor has a single gauge factor (GF, representing strain sensitivity) of 1.33 within the strain range of 0%–1300%. After the introduction of sorbitol and ChCl, the PLTAV‐SC sensor displays a linear relatively low GF of 0.23 in the strain range of 0%–1500%. When the strain exceeds this range, the GF increases to 0.77 due to changes in ion channels. At −45°C, the PLTAV‐SC sensor possesses three distinct GF (0.51, 1.39, and 3.29), as shown in Figure S35. From Figure 7b, the PLTAV and PLTAV‐SC sensors present the minimum detectable strains, which are 0.5% and 0.7%, respectively. Possibly owing to the excellent elasticity and ultralow hysteresis, the response (38; 19 ms) and recovery times (40; 21 ms) of PLTAV and PLTAV‐SC sensors are very short and close, and are superior to those of previously reported strain sensors, such as fully polymeric conductive hydrogel (P(AAm‐THMA‐ILs)/PP) sensor (65 and 40 ms) [24], semi‐interpenetrating poly(ionic liquid) hydrogel (PATV) sensor (180 and 190 ms) [44], and so on (more details are recorded in Table). As disclosed in Figure 7c,d, the electrical signals of the PLTAV and PLTAV‐SC sensors exhibit distinct differences at different strains. Moreover, the tensile frequency has no significant impact on the strain detection accuracy of the PLTAV and PLTAV‐SC sensors (Figure 7e) due to fast response and recovery. During repeated loading–unloading cycles, the electrical signal changes of the as‐prepared sensors remain stable (Figure 7f), which demonstrates their exceptional durability. In addition to detecting tensile stress based on tensile strain, the as‐prepared sensors can also accurately detect compressive stress. From Figure 7g and Figure S36a, the PLTAV and PLTAV‐SC sensors have ultrawide compressive stress detection ranges, with maximum detectable stress of 6.4 and 6.9 MPa, respectively. Their maximum sensitivities are 0.59 and 0.052 kPa^−1^, respectively. At −45°C, the PLTAV‐SC sensor maintains outstanding pressure sensing performance (Figure S36b). As illustrated in Figure S37, the as‐prepared sensors can reliably detect pulse signals, including three characteristic peaks: the advancing wave (P_1_), the reflection wave (P_2_), and the double beat wave (P_3_). Compared to previously reported strain sensors (Figure 7h) [24, 26, 48, 68, 69, 70, 71], the as‐prepared PLTAV and PLTAV‐SC sensors display ultrawide‐strain‐range linearity (0.5%–1300%; 0.7%–1500%) in resistance‐type strain sensing, and can be used for self‐powered and visual strain sensing simultaneously. Notably, the PLTAV‐SC sensor also has outstanding freezing resistance and can be applied under all‐weather conditions (more details are recorded in Table S5).

Benefiting from their outstanding sensing properties, the as‐prepared strain sensors have great potential for underwater applications. To avoid the impact of underwater ion exchange on the ionic conductivities of the strain sensors, which would result in the invalidity of the sensing performance, hydrophobic coatings are prepared for encapsulating the sensors, as shown in Figure S38. Since monomers (LMA and 2‐Ethylhexyl acrylate) of the hydrophobic coatings have good compatibility with hydrophobic microdomains of the sensors, these monomers can partially penetrate the sensor surface and form interpenetrating polymer networks after polymerization at the sensor‐coating interface, which ensures tight interfacial bonding. The resulting sensors are denoted as PLTAV‐H and PLTAV‐SC‐H sensors. As demonstrated in Figure S39, the static water contact angle of the sensor with the hydrophobic coating is much higher than those of the PLTAV and PLTAV‐SC sensors, which implies that the PLTAV‐H and PLTAV‐SC‐H sensors have excellent waterproofing capability. The sensor with the hydrophobic coating can adhere to various materials under water (Figure S40), which means that the PLTAV‐H and PLTAV‐SC‐H sensors possess good underwater adhesion performance. Furthermore, the as‐prepared PLTAV‐H and PLTAV‐SC‐H sensors retain excellent stretchability and flexibility (Figure S41). For practical applications, a wireless strain sensing system is constructed by integrating a signal collecting device (Figure S42) with a mobile phone (Figure 7i). Subsequently, the PLTAV‐H and PLTAV‐SC‐H sensors are attached to different body parts and monitor underwater real‐time signals (Figure 7i). As displayed in Figure 7j–l, the PLTAV‐H and PLTAV‐SC‐H sensors can detect various body movements, including finger bending, wrist bending, and elbow bending. Furthermore, they can detect pulse signals even in water (Figure 7m), which enables real‐time monitoring of the user's health status. Water snake (model) motion can also be monitored by attaching the as‐prepared sensors to one side (Figure 7n). As shown in Figure 7o, the swing amplitude of the water snake can be clearly identified based on differences in signal intensity. Intriguingly, the PLTAV‐H and PLTAV‐SC‐H sensors can detect water flow velocity (Figure 7p). As water flow velocity increases, the relative resistance of the sensors decreases (Figure 7q), and the detection upper limits exceed 9.6 L/min. Additionally, the immersed depth of these sensors in water can also be detected (Figure 7r). As illustrated in Figure 7s, these sensors exhibit different relative resistance values at varying water depths (0–6 m). This is attributed to changes in water pressure acting on the sensors. The above results suggest that the as‐prepared sensors have tremendous potential for application in aquatic environments.

## 3 Conclusion

In summary, an epithelium‐like structure hydrogel (PLTAV) has been successfully synthesized via a water‐in‐oil high internal phase emulsion. Benefiting from the hydrophobic microdomain and deformable cell‐like microgel particles, the as‐prepared PLTAV hydrogel with high water content has ultrahigh toughness, ultralow hysteresis, excellent stretchability, and ultrahigh compressibility. Subsequently, sorbitol and ChCl have been introduced into the PLTAV hydrogel. Owing to the salting‐out and hydration effects, the as‐prepared PLTAV‐SC hydrogel exhibits improved toughness, stretchability, and freezing resistance, along with retained low hysteresis and high compressibility. The difference in cationic and anionic mobilities and the AIE effect allow these hydrogels to act as self‐powered and visual strain sensors. Moreover, the PLTAV and PLTAV‐SC resistance‐type strain sensors possess exceptional properties (e.g., ultrawide‐strain‐range linearity and ultrashort response/recovery times) and have been further constructed as underwater strain sensors for various underwater applications (e.g., human/animal motion recognition, water flow velocity detection, and water depth detection). The presented work for the first time provides a biomimetic and facile strategy for the preparation of ultrahigh toughness, ultralow hysteresis, and highly compressible hydrogels and the construction of high‐performance and multi‐mode strain sensors.

## Conflicts of Interest

The authors declare no conflicts of interest.

## Supporting information




**Supporting File**: advs73587‐sup‐0001‐SuppMat.docx.

## Data Availability

The data that support the findings of this study are available from the corresponding author upon reasonable request.
