# Supplementary material for: Epithelium‐Inspired, Ultrahigh‐Toughness, Ultralow‐Hysteresis, and Highly Compressible Polymer Hydrogels as Self‐Powered, Visual, and Underwater Strain Sensors
Source: Adv Sci (Weinh). 2025 Dec 30;13(14):e10444. doi: 10.1002/advs.202510444 (PMC12970209; doi:10.1002/advs.202510444)
Supplement: Supplementary file 1 — Supporting File: advs73587‐sup‐0001‐SuppMat.docx. [file ADVS-13-e10444-s001.docx]

Supporting Information

**Epithelium-Inspired, Ultrahigh-Toughness, Ultralow-Hysteresis, and Highly Compressible Polymer Hydrogels as Self-Powered, Visual, and Underwater Strain Sensors**

*Yutang Zhou, Honghao Shu, Yuhuan Yao, Bolin Lu, and Wenbin Zhong**

Y. Zhou, H. Shu, Y. Yao, B. Lu, and Prof. W. Zhong

College of Materials Science and Engineering, Hunan University, Changsha 410082, China

E-mail: [wbzhong@hnu.edu.cn](mailto:wbzhong@hnu.edu.cn)

**Experimental Section**

**Materials**

Acrylamide (AM, 99%), 1-vinyl-3-butyl imidazole bromide (VBIBr, 99%), lauryl methacrylate (LMA, 96%), [2-(4-vinylphenyl)ethene-1,1,2-triyl] tribenzene (TPEE, 98%), Poly(ethylene glycol) diacrylate (PEGDA, Mw~1000), Phenylbis(2,4,6-trimethylbenzoyl)phosphine oxide (BAPO, 98%), 2-Hydroxy-2-methylpropiophenone (Photoinitiator 1173, 97%), Ethylene glycol dimethacrylate (EGDMA, 98%), 2-Ethylhexyl acrylate (EHA, 99%), Choline chloride (ChCl, 98%), and D-Sorbitol (Sorbitol, 98%) were purchased from Shanghai Macklin Biochemical Co., Ltd. (Shanghai, China). Span 80 (CP), N,N-Dimethylformamide (DMF, AR), Paraffin (AR), and Rhodamine B (AR) were obtained from Sinopharm Chemical Reagent Co., Ltd. (Shanghai, China). Double-distilled water was used in all experiments.

**Preparation of hydrogels**

*Synthesis of PLTAV hydrogel.* 0.2 g of Span 80, 1 mg of TPEE, and 20 mg of BAPO were dissolved in 2 g of LMA to form a hydrophobic precursor solution. Separately, 6 g of AM, 6 mg of VBIBr, and 3 mg of PEGDA were dissolved in 9 mL of water to form a hydrophilic precursor solution. Then, 1.5 mL of the hydrophilic precursor solution was added dropwise into 0.25 mL of the hydrophobic precursor solution at 50 °C. After stirring, a water-in-oil high internal phase emulsion was obtained. This emulsion was injected between two glass sheets separated by a 1 mm-thick silicone film and polymerized under white light (10 W) for one hour. After polymerization, the sample was removed from the mold and soaked in water until its mass stabilized. The resulting hydrogel was denoted as the PLTAV hydrogel. The composition of the PLTAV hydrogel was optimized by adjusting the volume ratio of the hydrophobic precursor solution to the hydrophilic precursor solution, the type and dosage of crosslinking agent, the initiator dosage, and the solid content of the hydrophilic precursor solution (Tables S1 and S2).

*Synthesis of SE hydrogel.* By stirring, AM (6 g), VBIBr (6 mg), PEGDA (3 mg), and the hydrophobic precursor solution (1.8 g) were dissolved in DMF (40 g) to form a homogeneous solution. Then, the solution was injected into the mold and polymerized under the same conditions described above. Subsequently, it was transferred from the mold and soaked in water to remove DMF until its mass stabilized. The resulting hydrogel exhibited a water content comparable to that of the PLTAV hydrogel and was denoted as the SE hydrogel, named after the solvent exchange method.

*Synthesis of PAV hydrogel.* 15 mg of Photoinitiator 1173 was dissolved in the hydrophilic precursor solution and stirred to form a homogeneous solution. Then, the solution was injected into the mold and polymerized under UV light (10 W) for one hour. Afterward, it was transferred from the mold and soaked in water until its water content matched that of the PLTAV hydrogel. The resulting hydrogel was denoted as the PAV hydrogel.

*Preparation of PLTAV-SC hydrogel.* The PLTAV hydrogel was immersed in a mixed aqueous solution of 2 M sorbitol and 4 M ChCl for 48 hours. The resulting hydrogel was denoted as the PLTAV-SC hydrogel.

**Preparation of hydrophobic coating**

The process for preparing hydrophobic coatings of hydrogels was similar to that reported in the previous literature.^[1]^ Specifically, 2.5 mL of LMA, 2.8 mL of EHA, 14 uL of EGDMA, and 20 mg BAPO were added to 2.4 mL of paraffin and stirred thoroughly. Then, the as-prepared hydrogel (length (L): 30 mm; width (W): 10 mm) was placed in a self-made mold composed of two glass sheets and one silicone film. By varying the height of the silicone film, the thickness of the hydrophobic coating was controlled. Next, the precursor was rapidly injected into the mold and immediately polymerized under white light (10 W). After that, the mold was replaced, and the same procedure was repeated to construct the hydrophobic coating.

**Mechanical tests**

The tensile tests were performed on hydrogel samples (L: 40 mm; W: 10 mm) using a CMT6103 (MTS Systems Ltd., America) at an extension rate of 30 mm/min. The modulus and toughness of each sample were determined by calculating the slope within the 0-10% strain range and the area under the curve. The compressive stress-strain curves of hydrogels (Height: 8 mm) were obtained using an AGX-V 50 kN (Shimadzu Ltd., Japan) at a compressive rate of 1 mm/min. The tensile and compressive stress-strain curves of the hydrogel samples during loading-unloading processes were measured under identical conditions. The hysteresis ratios (H) were calculated by following the formula:

$$H=\left( A_{loading}-A_{unloading} \right)/{A_{loading}}\times100\%$$

where A_loading_ and A_unloading_ were the areas of loading and unloading curves, respectively.

**Density functional theory calculations**

All calculations were performed using Gaussian 16. The B3LYP hybrid functional at 6-31g* level of basis set involving the atom-pairwise dispersion (DFT-D3) correction with Becke-Johnson (BJ) damping was adopted for geometry optimizations. Single-point energies were calculated at B3LYP/def2-TZVP including the DFT-D3 (BJ) correction. The binding energy (E) was obtained using the following equation:

$$E=E_{AB}-\left( E_{A}+E_{B} \right)$$

where E_AB_, E_A_, and E_B_ were the single-point energies of AB complex, A, and B, respectively. The value of E reflected the interaction between two molecules. A smaller E meant a stronger intermolecular interaction.

**Ionic conductivity tests**

The ionic conductivities (σ) of the as-prepared hydrogels were obtained according to the formula:

$$\sigma=L/{(R\times S)}$$

where L, R, and S were the length, resistance, and cross-sectional area of each sample, respectively. The R was measured by CHI660E (CH Instruments Ltd., America) using a two-probe method.

**Real-time relative resistance detections**

The real-time resistance of each hydrogel was recorded using either a CHI660E electrochemical workstation or a wireless strain sensing system. The relative resistance variation (ΔR/R_0_) could be acquired by the equation:

$${\Delta R}/{R_{0}} (\%)={{(R}_{t}-R_{0})}/{R_{0}}\times100\%$$

where R_t_ and R_0_ were the real-time resistance and original resistance of each sample, respectively.

**Other characterizations**

The chemical structure was studied using FTIR spectroscopy (Nicolet iS10 spectrometer, America) and XPS spectroscopy (Thermo ESCALAB 250Xi instrument, America). The morphology was investigated by OM (Olympus, Japan), CLSM (LSM980, Germany), SAXS (Xeuss 2.0, France), and AFM/KPFM (MFP-3D Origin, America). The rheological properties were measured using an MCR 302e rheometer (Anton Paar Ltd., Austria). The absorbance was measured using a UV-2550 (Shimadzu Ltd., Japan). The freezing resistance was evaluated using a DSC 200 F1 (Netzsch, Germany). The real-time voltage signal was collected using an MT-1820 (Prokit's Industries Ltd., China). The fluorescence intensity was determined using an FP-8500 (JASCO, Japan). The adhesive performance was evaluated using an electronic dynamometer (HLB-500, China), and the ratio of maximum load to initial adhesion area was defined as the adhesive strength. To perform the fluorescence intensity measurement of as-prepared hydrogels, the as-prepared hydrogel with different tensile strain was fixed on a glass sheet, and then the glass sheet was placed in a quartz sampling cell for testing. When the sensing performance of hydrogel was measured, the environmental humidity was constant (54% RH).

**Informed Consent Statement**

All participants voluntarily agreed to participate in the wearable device experiment and provided written informed consent before any experimental procedure began. In addition, it was confirmed that the use of wearable devices for research does not require the approval of an institutional review board.

**Supporting Figures and Tables**


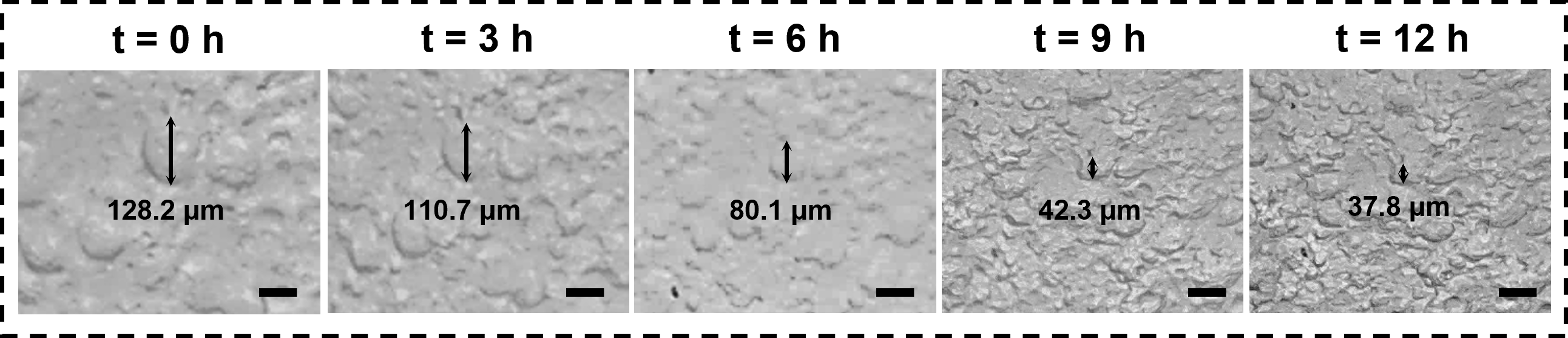


**Figure S1.** In-situ OM images of PLTAV hydrogel during drying. (Scale bar: 70 μm.)


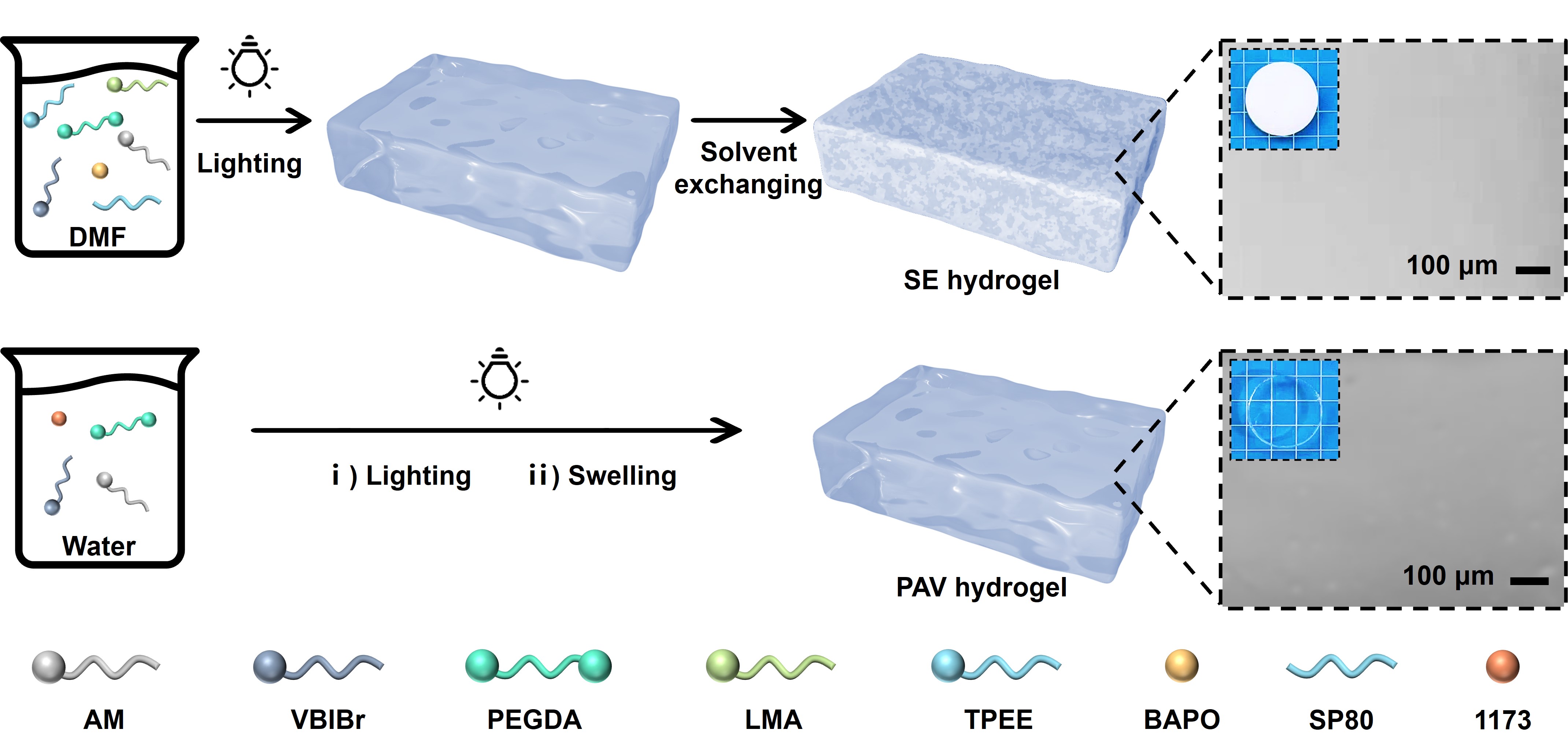


**Figure S2.** Preparation processes and OM images of SE and PAV hydrogels.

**Figure S3.** Ionic conductivity of PLTAV, SE, and PAV hydrogels

For the PLTAV hydrogel, the hydrophobic microdomains are distributed outside hydrophilic cell-like particles. The particles can form conductive channels and promote ion transport under an applied electric field. Thus, the ionic conductivity of the PLTAV hydrogel is higher than that of the PAV hydrogel (Figure S3). As for the SE hydrogel, the hydrophobic microdomains are irregularly distributed in the hydrogel. The hydrophobic microdomain hinders ion migration under an applied electric field. Therefore, the ionic conductivity of the SE hydrogel is lower than that of the PAV hydrogel.

**Figure S4.** FTIR spectra of PLTAV, SE, and PAV hydrogels.


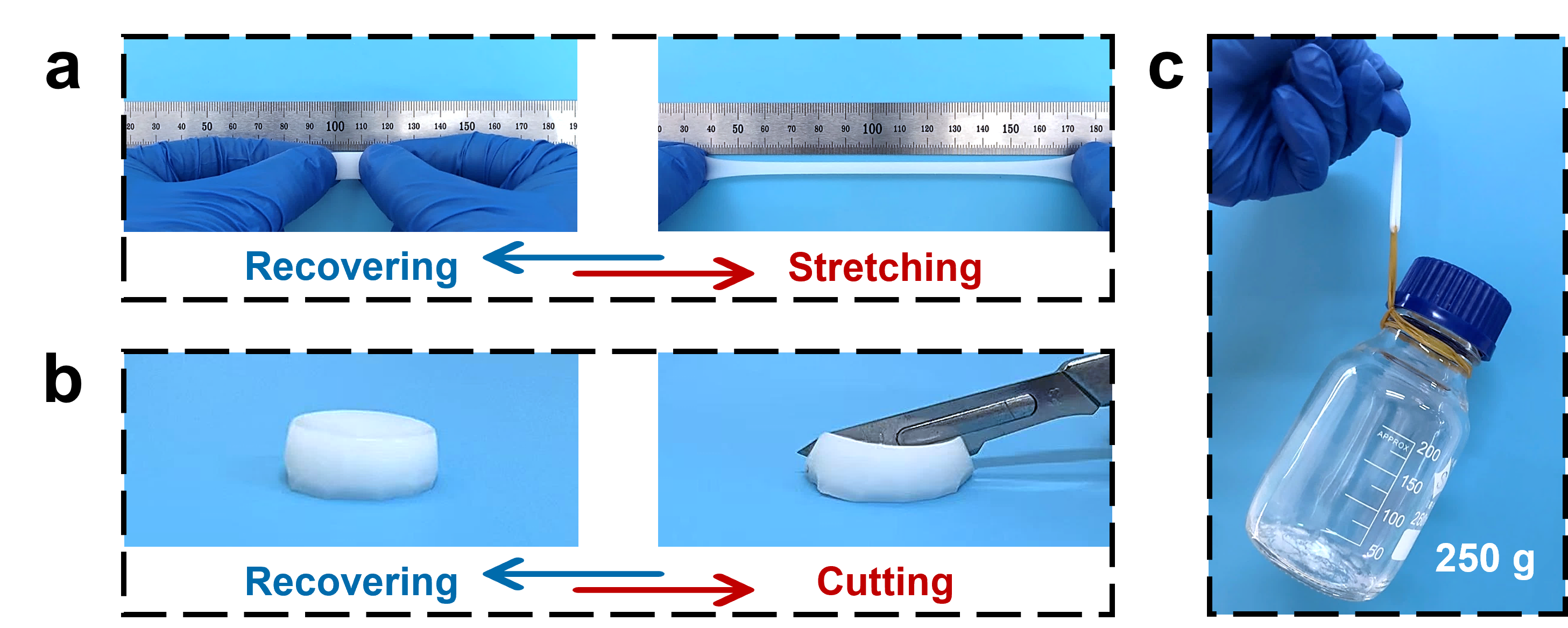


**Figure S5.** a) Deformability, b) cutting resistance, and c) weight bearing of PLTAV hydrogel.


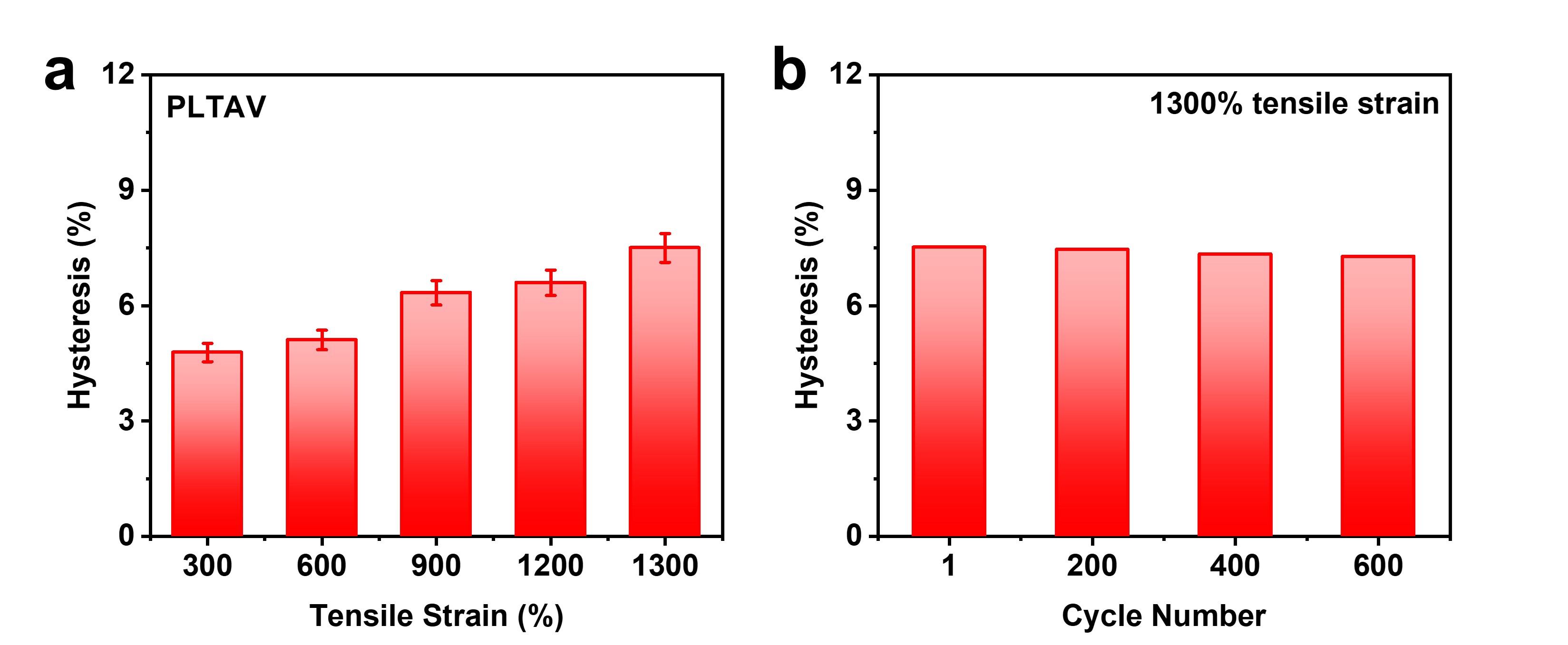


**Figure S6.** a) Hysteresis of PLTAV hydrogel at different tensile strains. b) Hysteresis of PLTAV hydrogel at a 1300% tensile strain after different cycles.


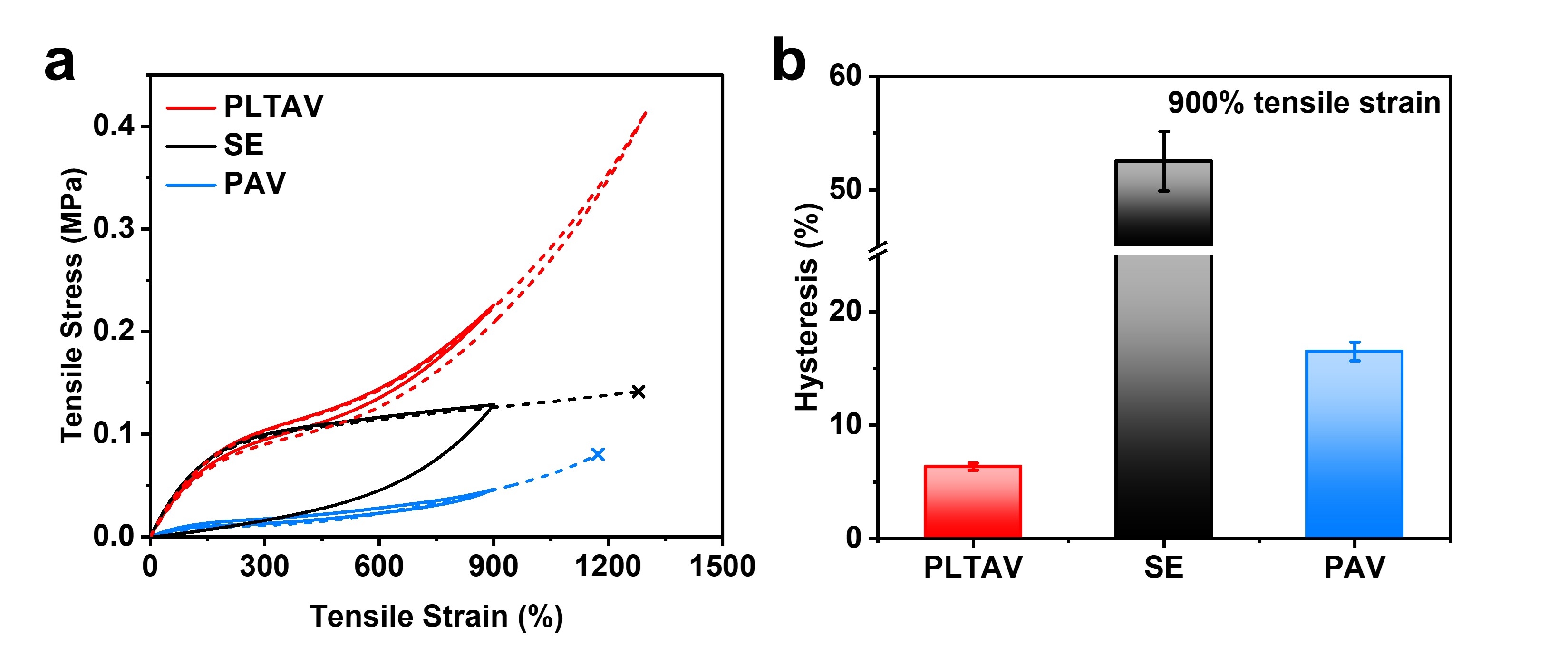


**Figure S7.** a) Tensile stress-strain curves of PLTAV, SE, and PAV hydrogels during loading-unloading processes at a 900% strain. b) Hysteresis of PLTAV, SE, and PAV hydrogels at a 900% tensile strain.


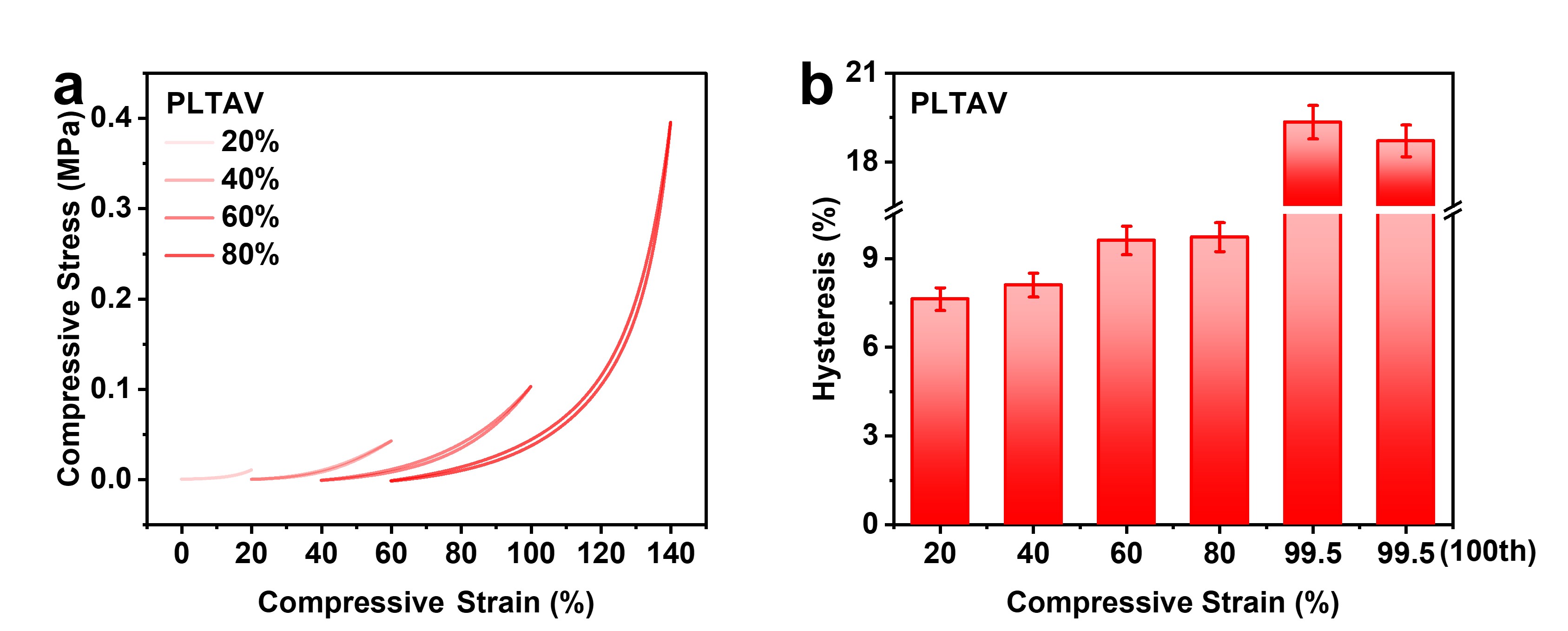


**Figure S8.** a) Compressive stress-strain curves of PLTAV hydrogel during loading-unloading processes at different strains. b) Hysteresis of PLTAV hydrogel at different compressive strains.


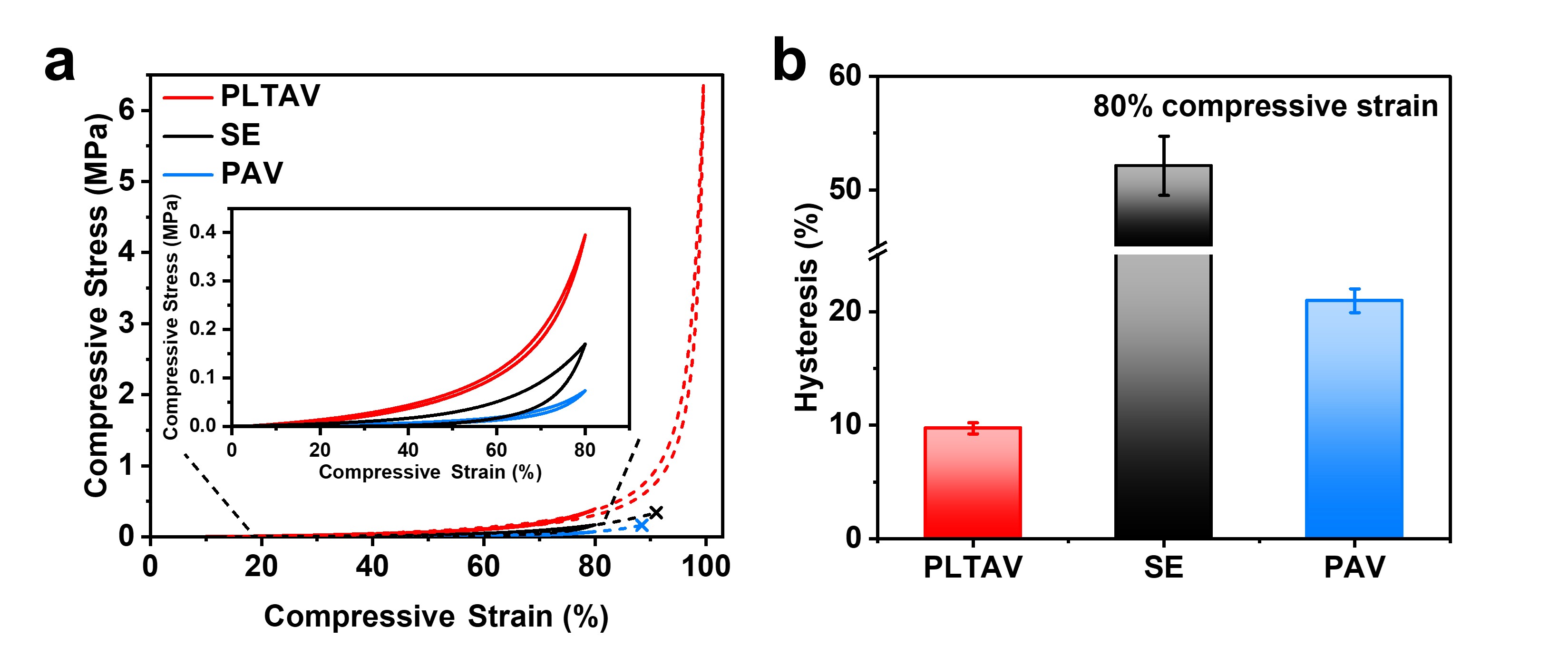


**Figure S9.** a) Compressive stress-strain curves of PLTAV, SE, and PAV hydrogels during loading-unloading processes at an 80% strain. b) Hysteresis of PLTAV, SE, and PAV hydrogels at an 80% compressive strain.


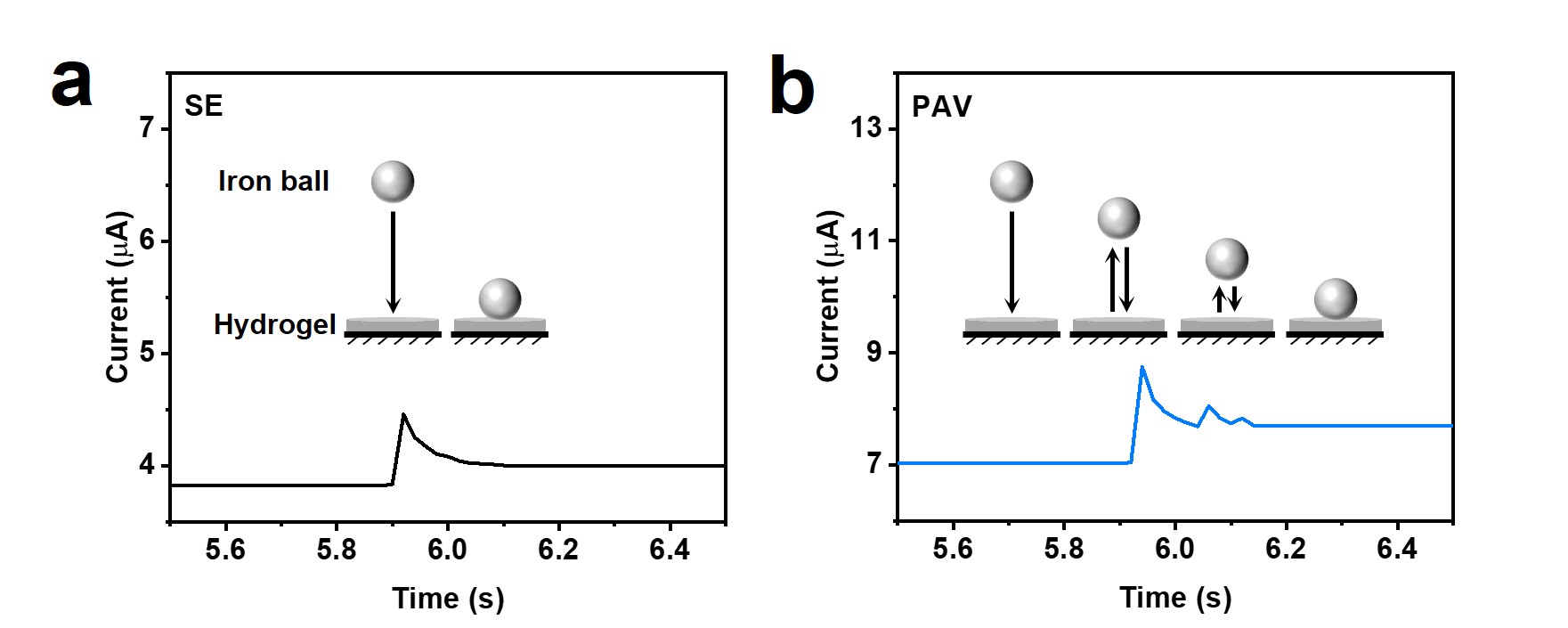


**Figure S10.** Real-time current changes of a) SE and b) PAV hydrogels during the impact of a falling iron ball (Weight: 5g; Initial height: 15 cm).


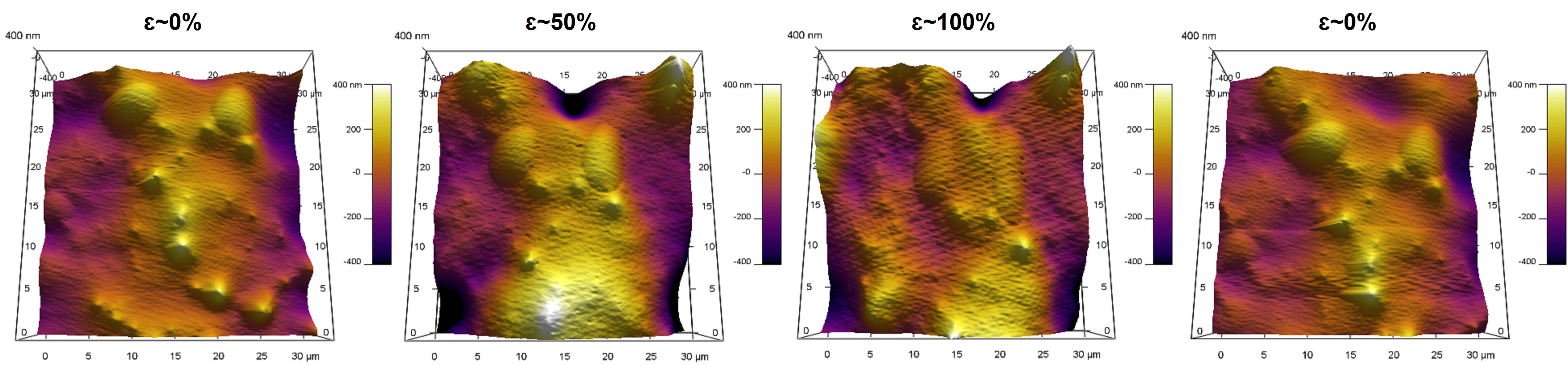


**Figure S11.** In-situ 3D AFM height images of PLTAV hydrogel during stretching and recovering.


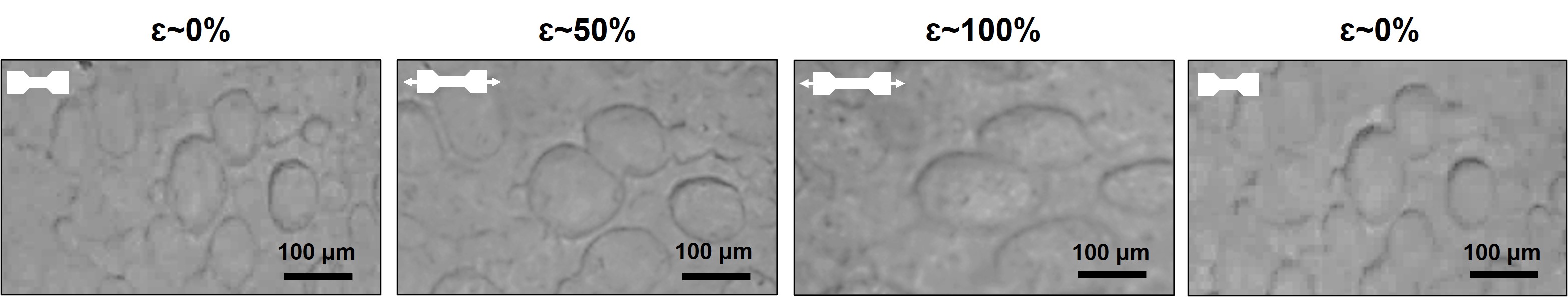


**Figure S12.** In-situ OM images of PLTAV hydrogel during stretching and recovering.


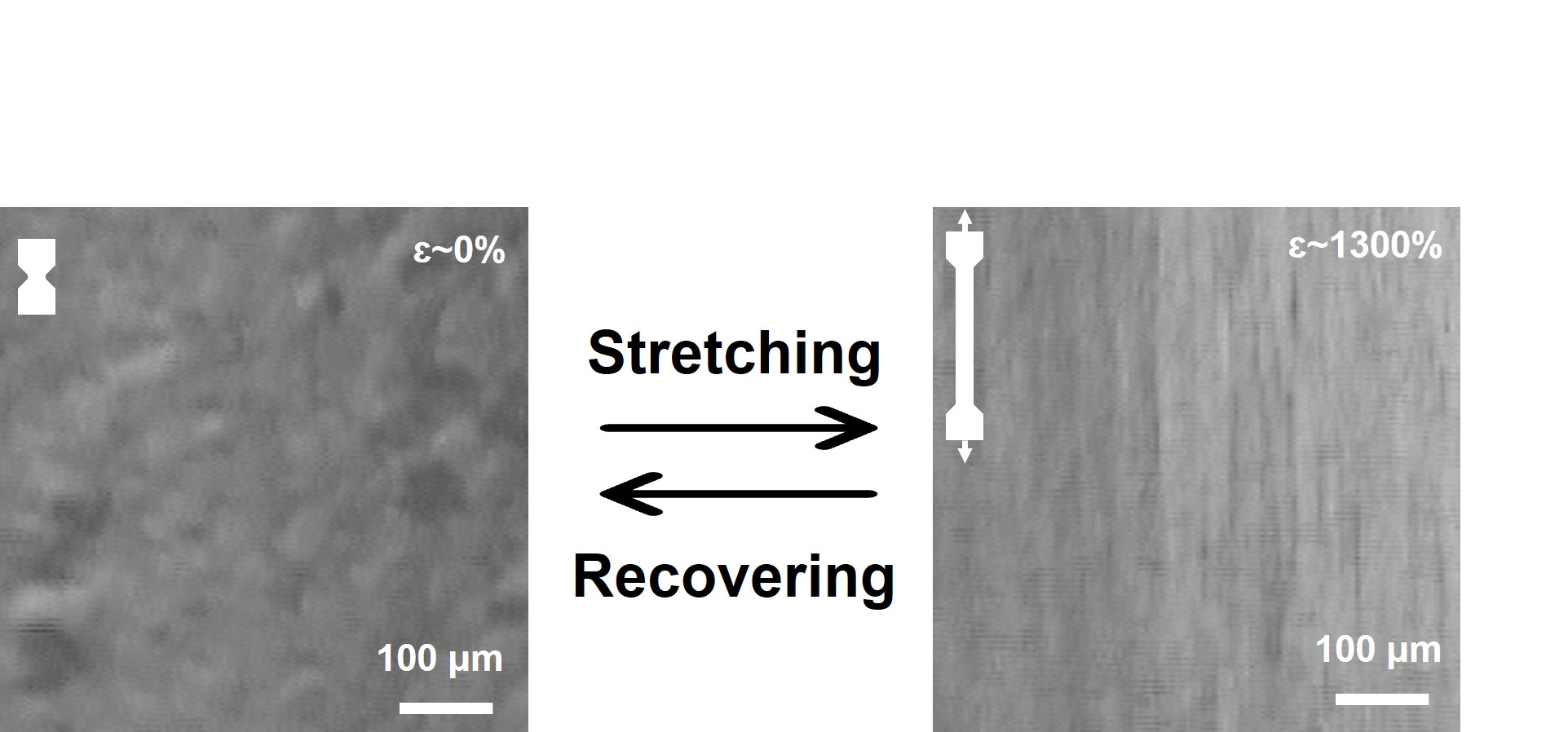


**Figure S13.** AFM optical images of PLTAV hydrogel at tensile strains of 0% and 1300%.


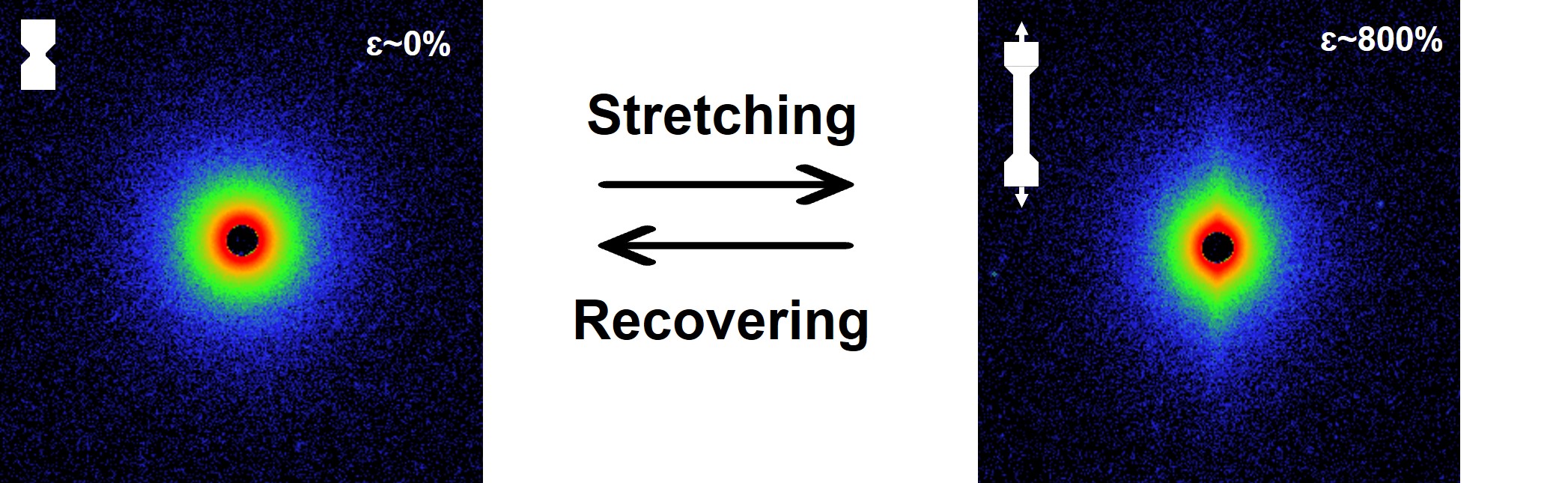


**Figure S14.** SAXS images of PLTAV hydrogel at tensile strains of 0% and 800%.


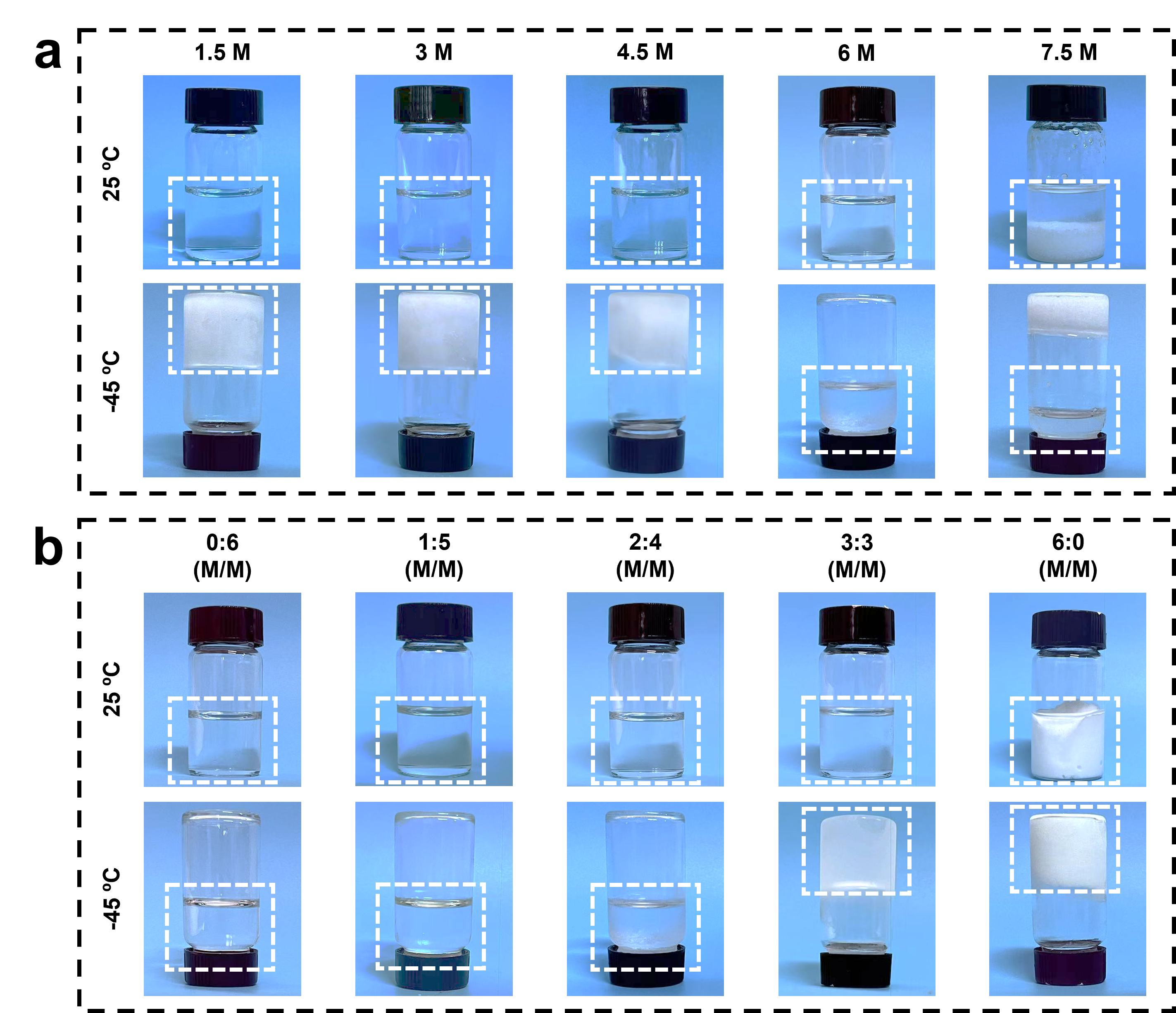


**Figure S15.** a) Digital images of solutions with different total concentrations at 25 ºC and -45 ºC (Molar concentration ratio of sorbitol to ChCl: 2:4). b) Digital images of solutions with different molar concentration ratios of sorbitol to ChCl at 25 ºC and -45 ºC (Total concentration: 6 M).

From Figure S15a, when the total concentration increases to 6 M, the mixed solution of ChCl and sorbitol becomes saturated and does not freeze at -45 ºC. As seen in Figure S15b, when the molar concentration ratios of sorbitol to ChCl are 0:6, 1:5, and 2:4, the mixed solutions do not freeze at -45 ºC. Therefore, 6 M mixed solutions with 0:6, 1:5, and 2:4 molar concentration ratios of sorbitol to ChCl were chosen to prepare PLTAV-SC hydrogels.


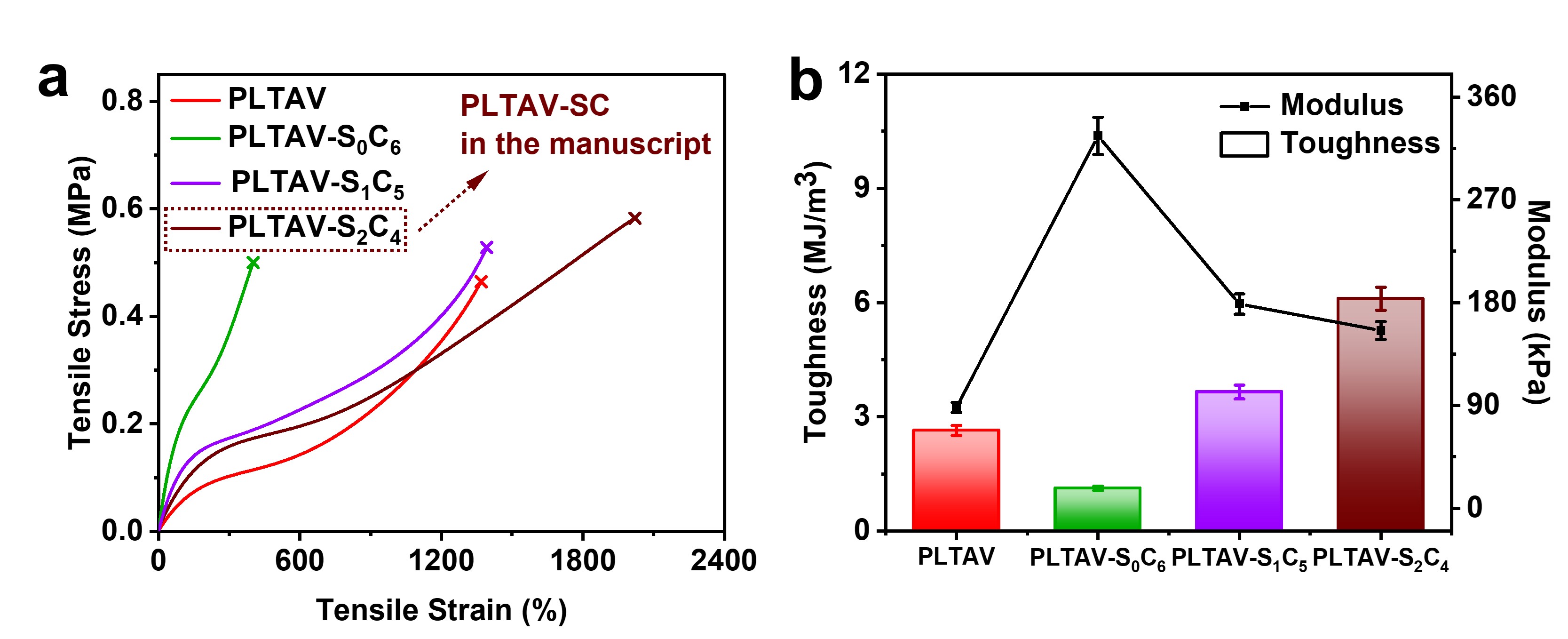


**Figure S16.** a) Tensile stress-strain curves of PLTAV-SC hydrogels with different Sorbitol and ChCl concentrations. b) Toughness and modulus of PLTAV-SC hydrogels with different Sorbitol and ChCl concentrations.

Figure S16a shows that ChCl renders the PLTAV hydrogel hard and brittle due to its strong salting-out effect. The addition of sorbitol weakens the effect of ChCl, which enhances the toughness of the PLTAV hydrogel (Figure S16b). When the molar concentration ratio of sorbitol to ChCl is 2:4, the resulting PLTAV-SC hydrogel exhibits the highest tensile strength, tensile strain, and toughness. Hence, this mixed solution was chosen to prepare the PLTAV-SC hydrogel.


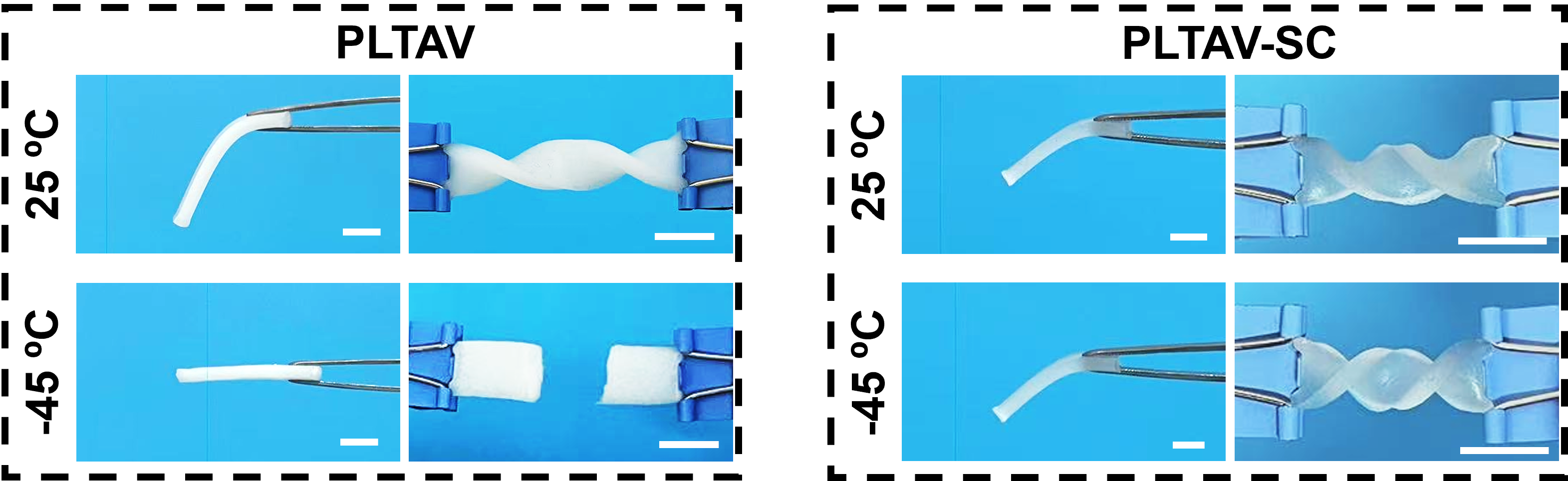


**Figure S17.** Flexibility of PLTAV and PLTAV-SC hydrogels at different temperatures.

**Figure S18.** FTIR spectra of PLTAV and PLTAV-SC hydrogels.

The FTIR spectra of the PLTAV and PLTAV-SC hydrogels are shown in Figure S18. After the introduction of sorbitol and ChCl, the characteristic peaks of N-H (3340 and 3193 cm^-1^) and C=O (1649 cm^-1^) of the PLTAV hydrogel shift to 3328, 3188, and 1647 cm^-1^, respectively, which indicates the formation of more hydrogen bonds.

**Figure S19.** XPS spectra of PLTAV and PLTAV-SC hydrogels.

From the XPS spectra (Figure S19), after introducing the mixed solution of sorbitol and ChCl, a new peak appears at 198 eV in the PLTAV-SC hydrogel, corresponding to Cl 2p, which suggests the successful incorporation of the mixed solution.


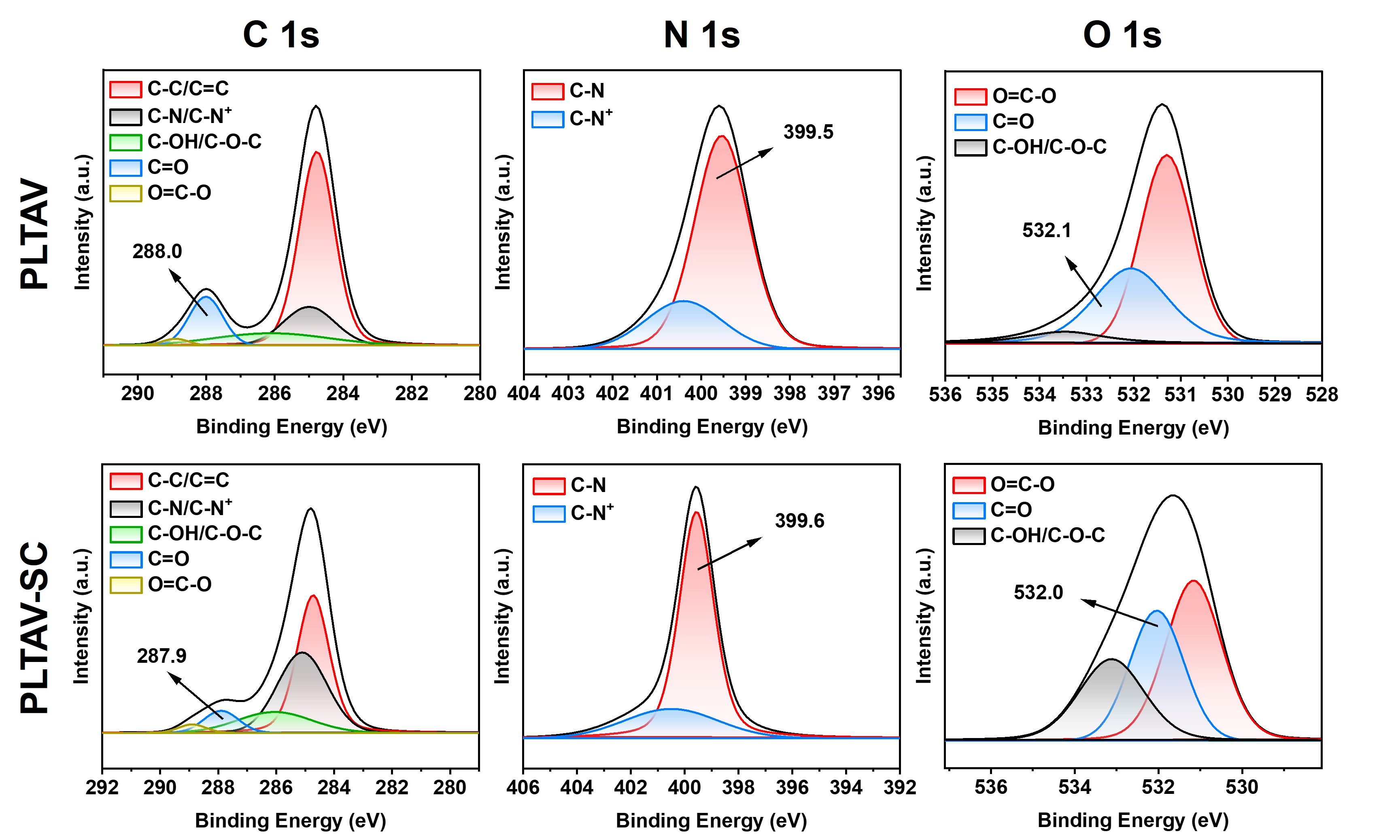


**Figure S20.** XPS C 1s, N 1s, and O 1s spectra of PLTAV and PLTAV-SC hydrogels.

The XPS C 1s, N 1s, and O 1s spectra of the PLTAV and PLTAV-SC hydrogels are presented in Figure S20. In the XPS C 1s spectra, the C=O peak of the PTLAV-SC hydrogel (287.9 eV) is lower than that of the PLTAV hydrogel (288.0 eV). In the XPS N 1s spectra, the binding energy of C-N in the PLTAV hydrogel increases from 399.5 to 399.6 eV after the introduction of sorbitol and ChCl. In the XPS O 1s spectra, the binding energy of C=O in the PTLAV-SC hydrogel is 532.0 eV, lower than that in the PLTAV hydrogel (532.1 eV). These results demonstrate that ChCl and sorbitol promote the formation of more hydrogen bonds among hydrophilic polymer chains.


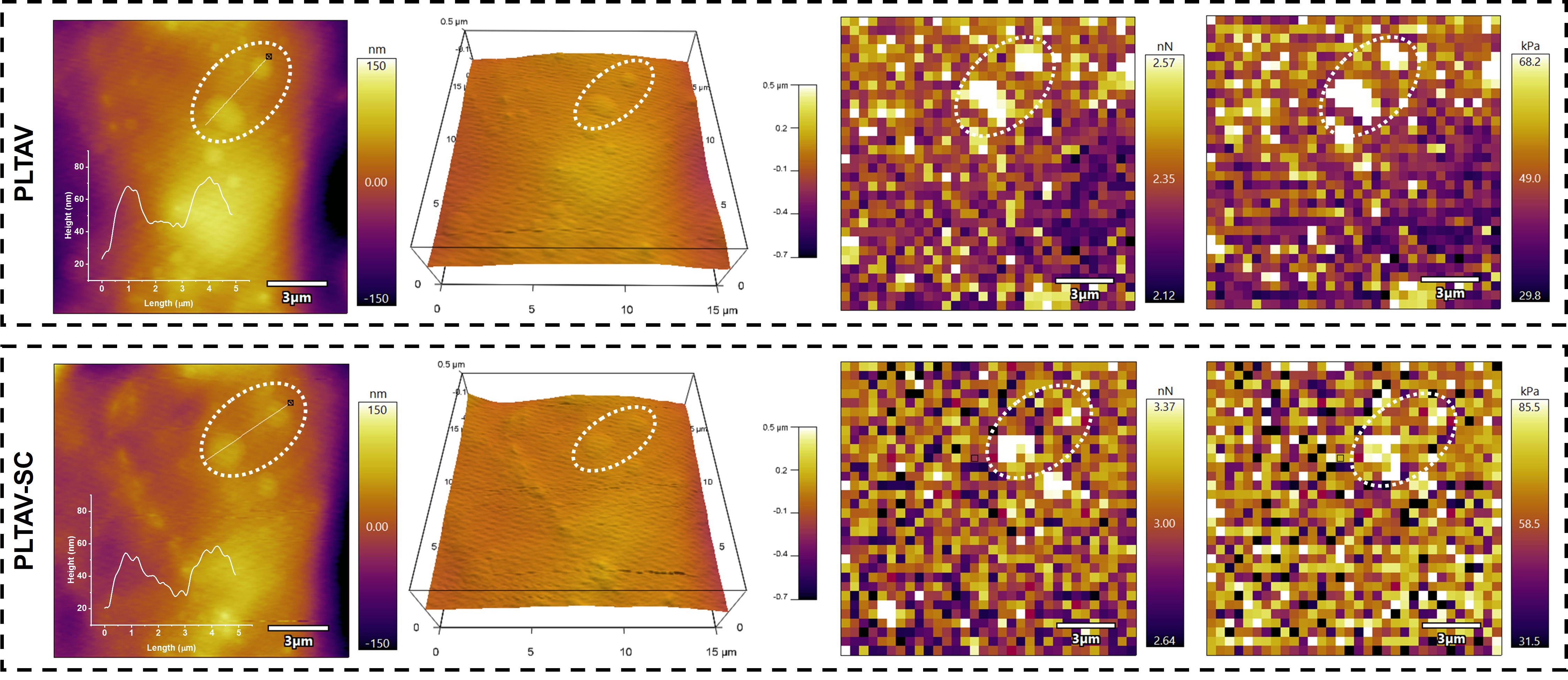


**Figure S21.** In-situ AFM height, adhesion, and modulus images of PLTAV and PLTAV-SC hydrogels.


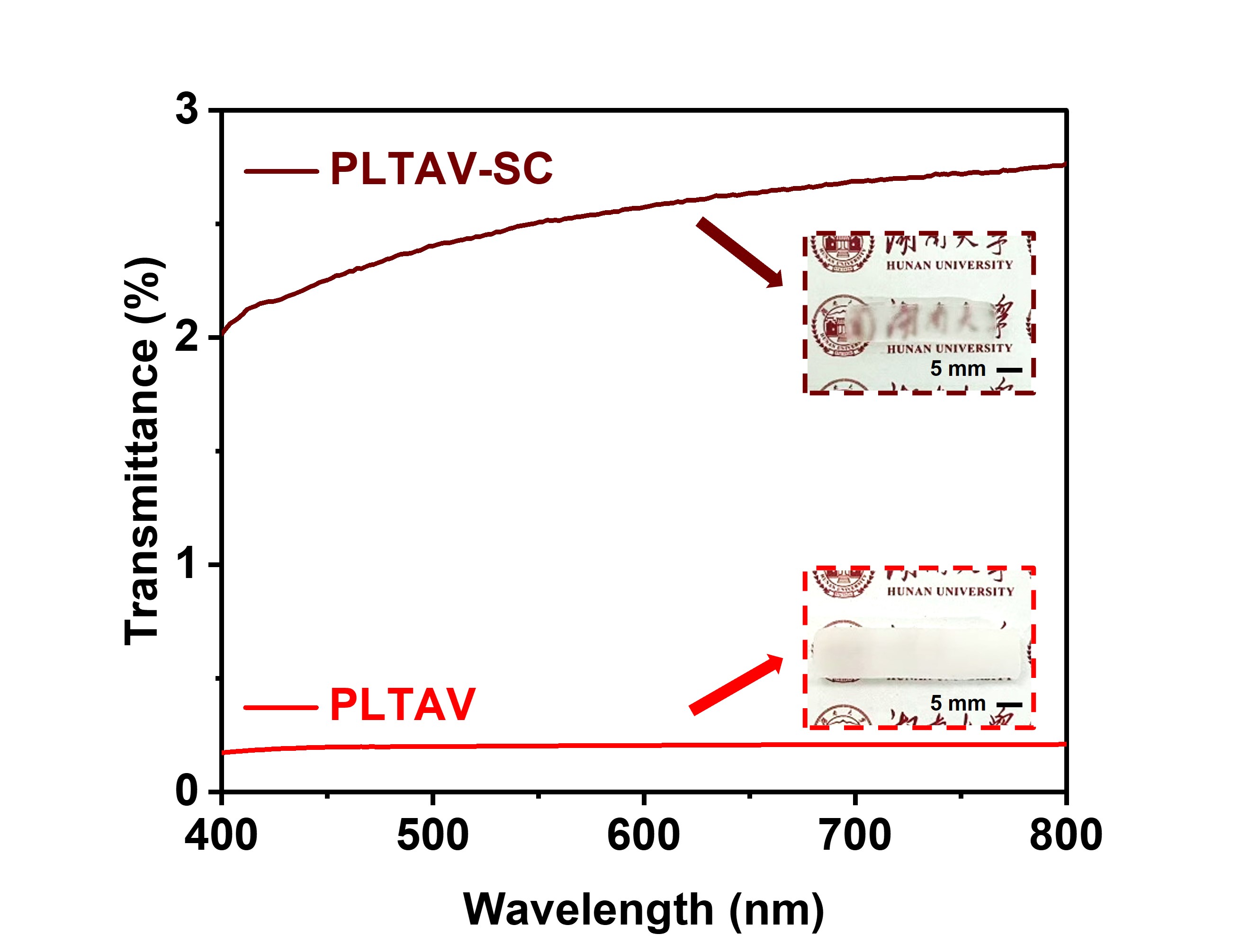


**Figure S22.** Transmittance of PLTAV and PLTAV-SC hydrogels in the visible light region.

**Figure S23.** Hysteresis of PLTAV-SC hydrogel at different tensile strains.


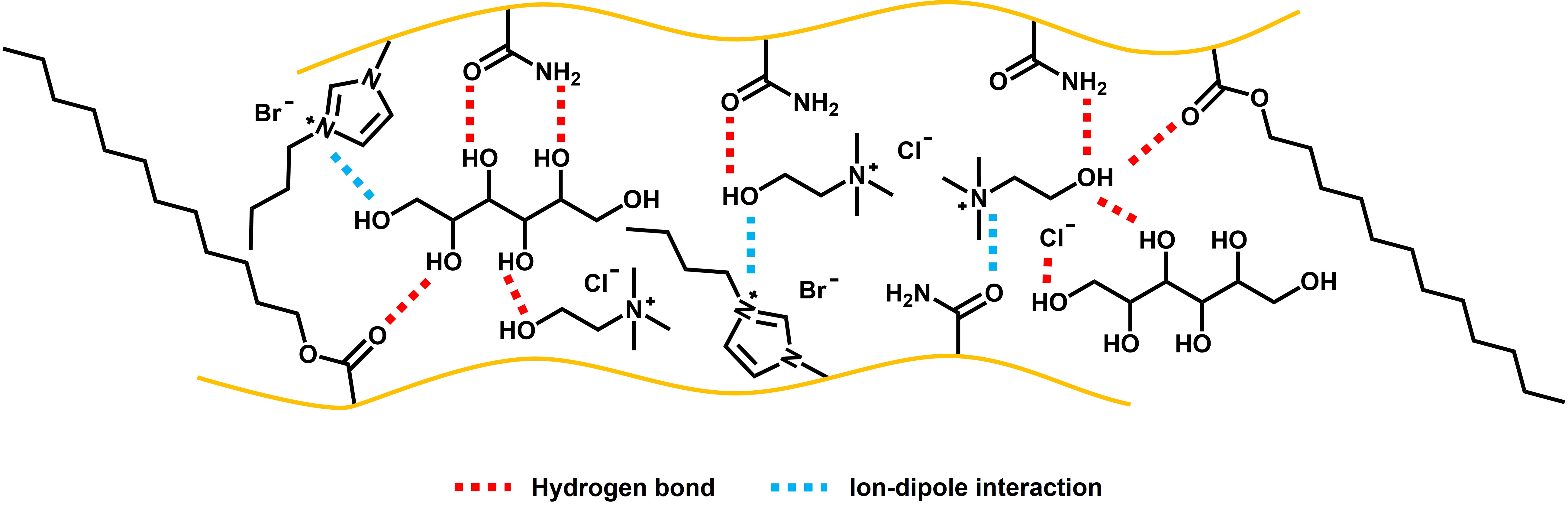


**Figure S24.** Interaction mechanism among sorbitol, choline chloride, and the polymer network.

As shown in Figure S24, with the introduction of sorbitol and choline chloride, the amide, imidazole, and ester groups of the polymer chains form many interactions with them such as hydrogen bonds and ion-dipole interaction.


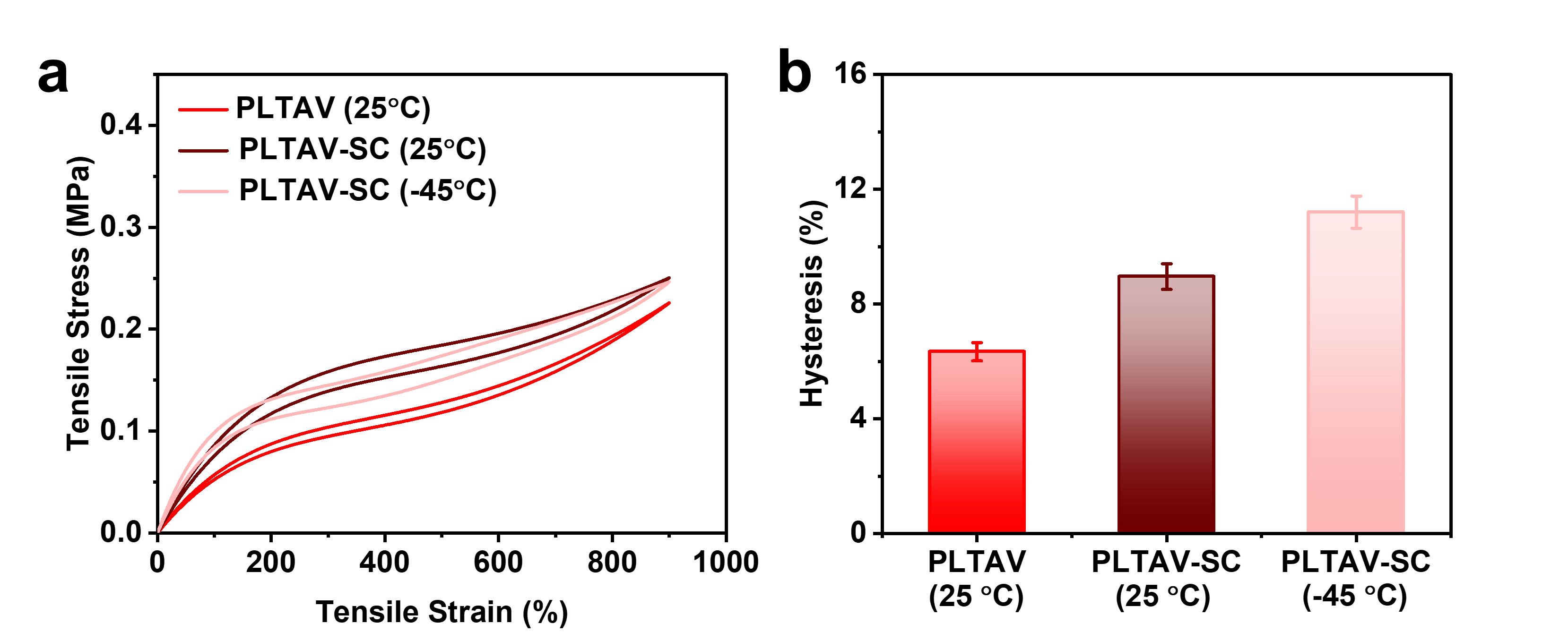


**Figure S25.** a) Tensile stress-strain curves of PLTAV and PLTAV-SC hydrogels during loading-unloading processes at a 900% strain at different temperatures. b) Hysteresis of PLTAV and PLTAV-SC hydrogels at a 900% tensile strain at different temperatures.


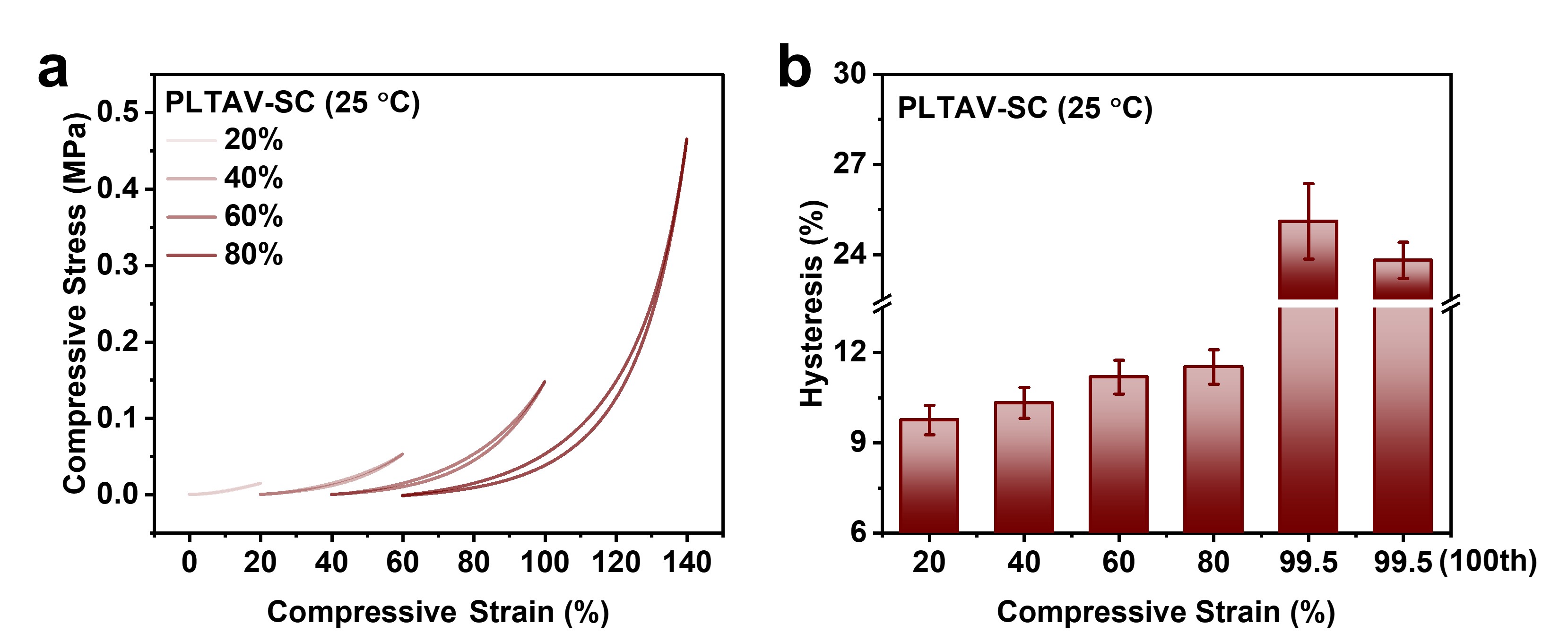


**Figure S26.** a) Compressive stress-strain curves of PLTAV-SC hydrogel during loading-unloading processes at different strains. b) Hysteresis of PLTAV-SC hydrogel at different compressive strains.


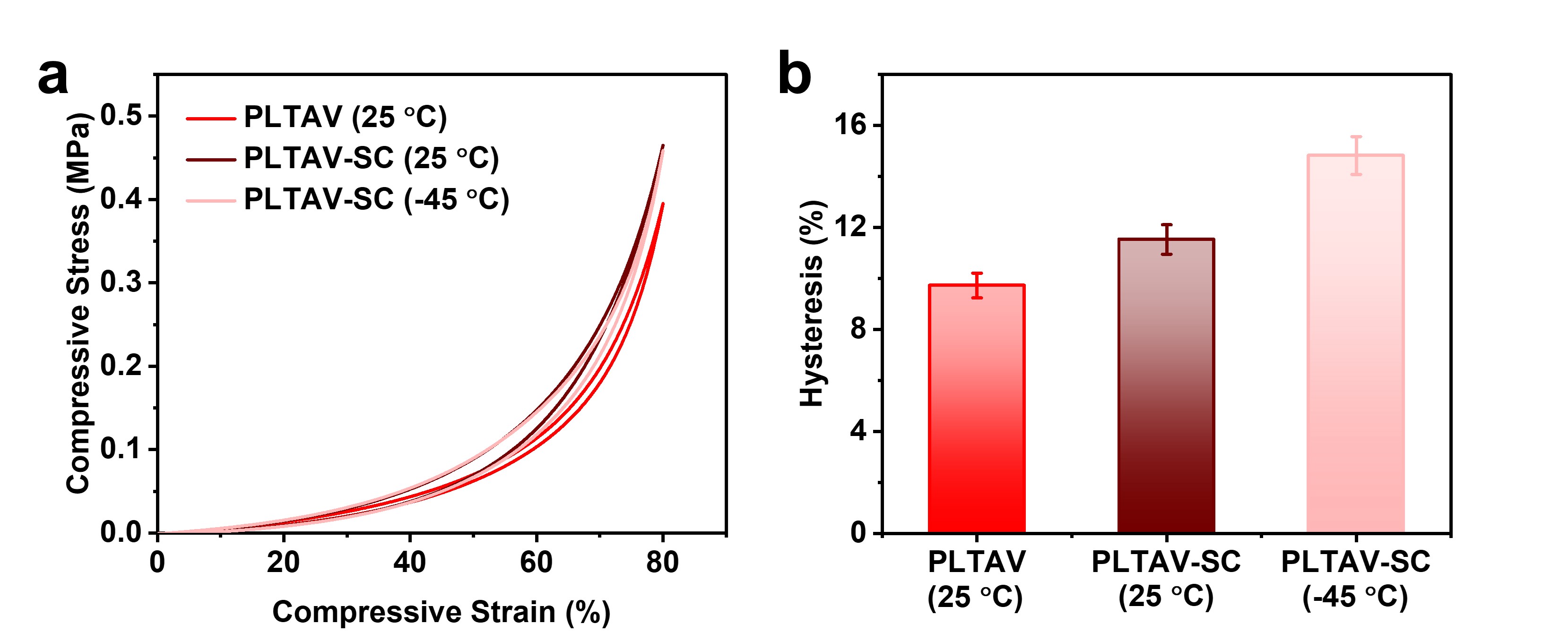


**Figure S27.** a) Compressive stress-strain curves of PLTAV and PLTAV-SC hydrogels during loading-unloading processes at an 80% strain at different temperatures. b) Hysteresis of PLTAV and PLTAV-SC hydrogels at an 80% compressive strain at different temperatures.


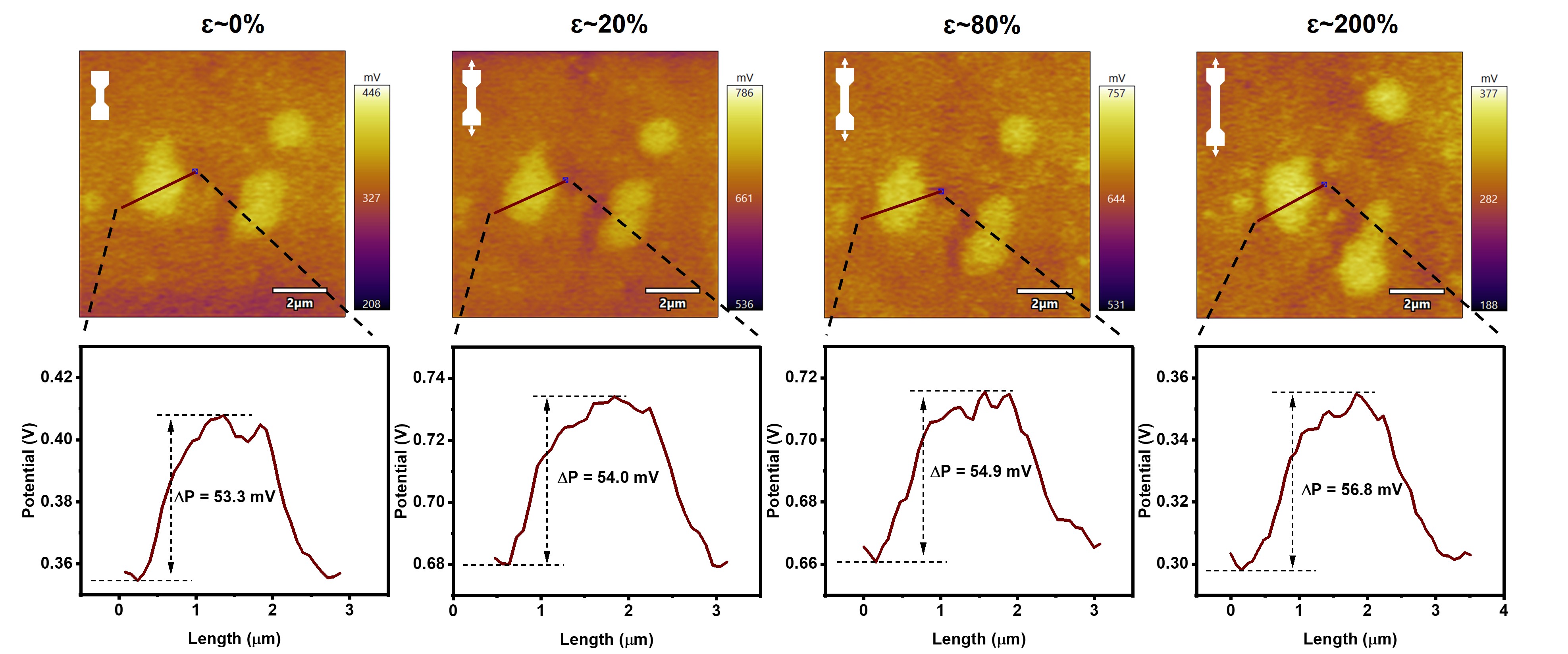


**Figure S28.** In-situ KPFM surface potential images of PLTAV-SC hydrogel during stretching.


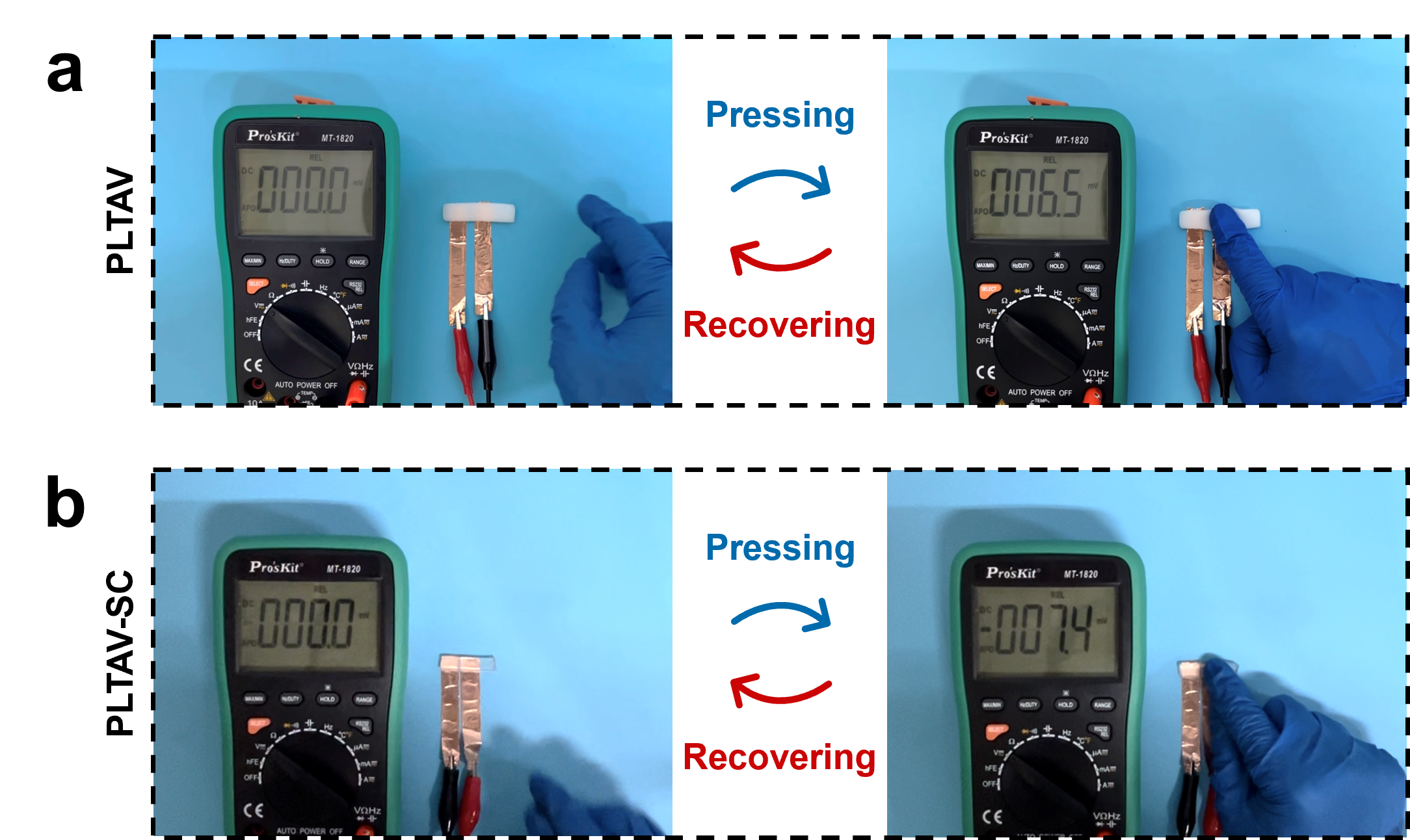


**Figure S29.** Digital images of PLTAV and PLTAV-SC hydrogels for piezoionic sensors.

**Figure S30.** Generated voltages of PLTAV-SC sensor with different compressive strains at -45 ºC.

**Figure S31.** Generated voltages of PLTAV, SE, and PAV sensors at a 40% compressive strain.


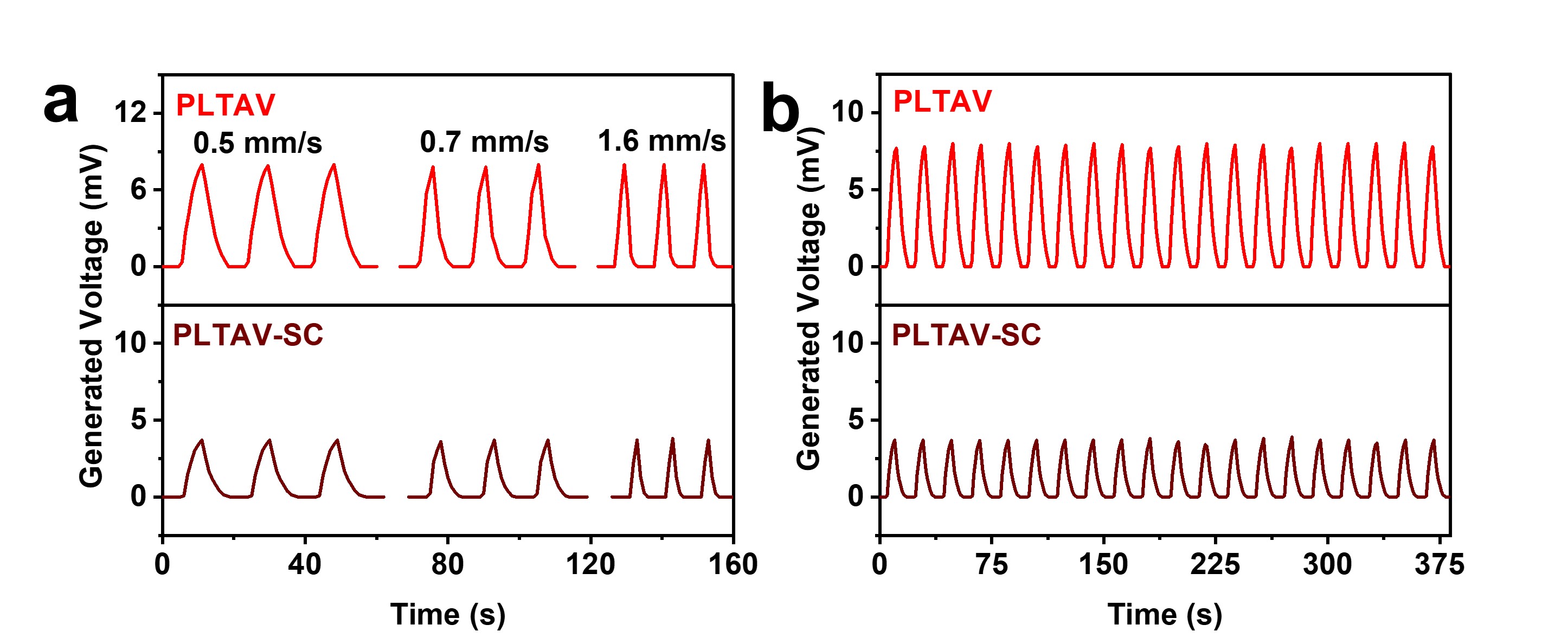


**Figure S32.** a) Generated voltages of PLTAV and PLTAV-SC sensors at a 40% compressive strain at different compressive speeds. b) Generated voltages of PLTAV and PLTAV-SC sensors during loading-unloading cycles at a 40% compressive strain.


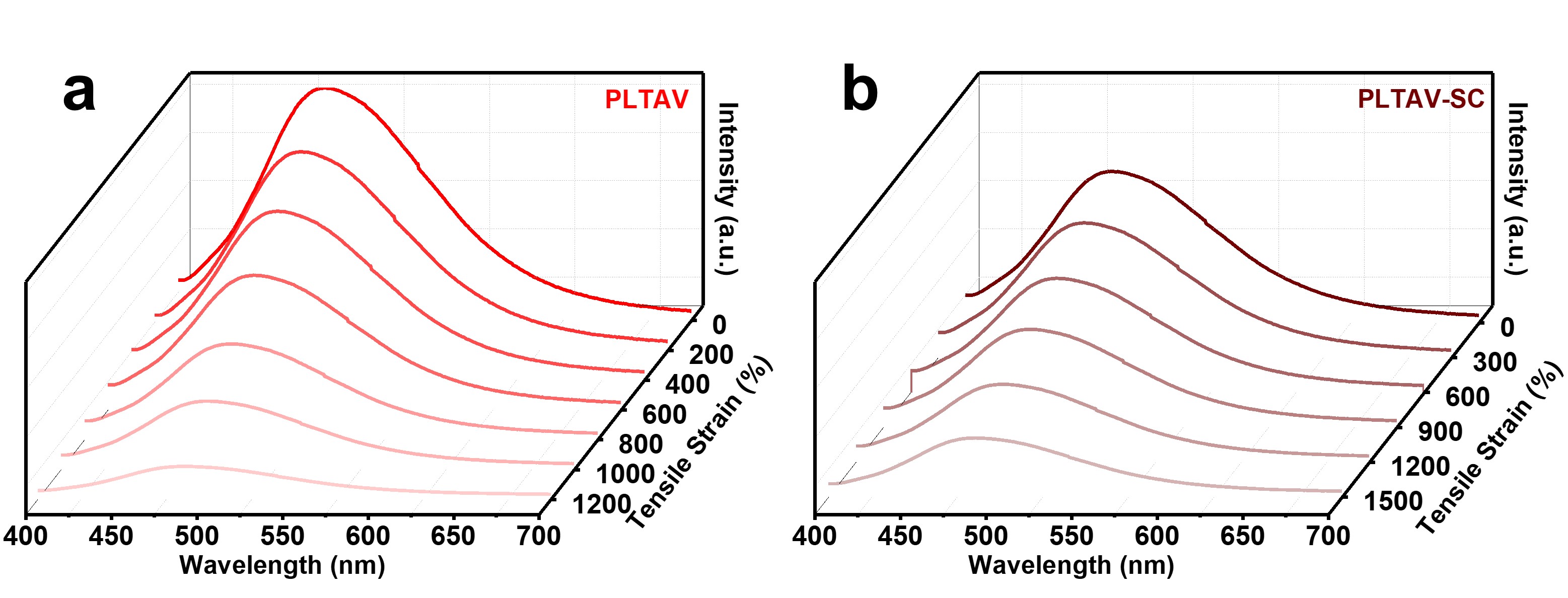


**Figure S33.** Fluorescence emission spectra of a) PLTAV and b) PLTAV-SC hydrogels with different tensile strains.


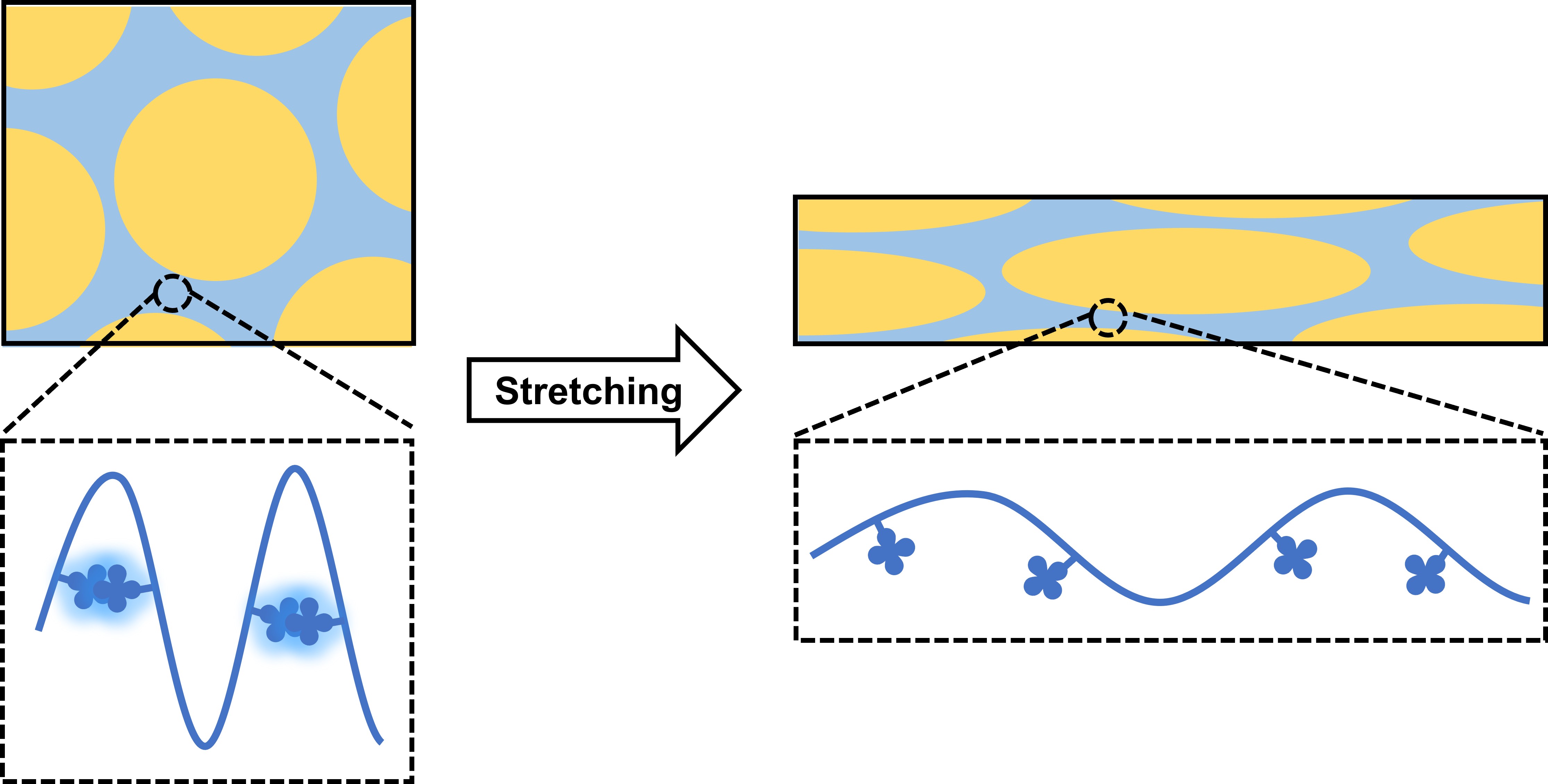


**Figure S34.** Schematic illustration of weakening of the AIE effect.

**Figure S35.** Relative resistance variation of PLTAV-SC hydrogel with tensile strain at -45 ºC.


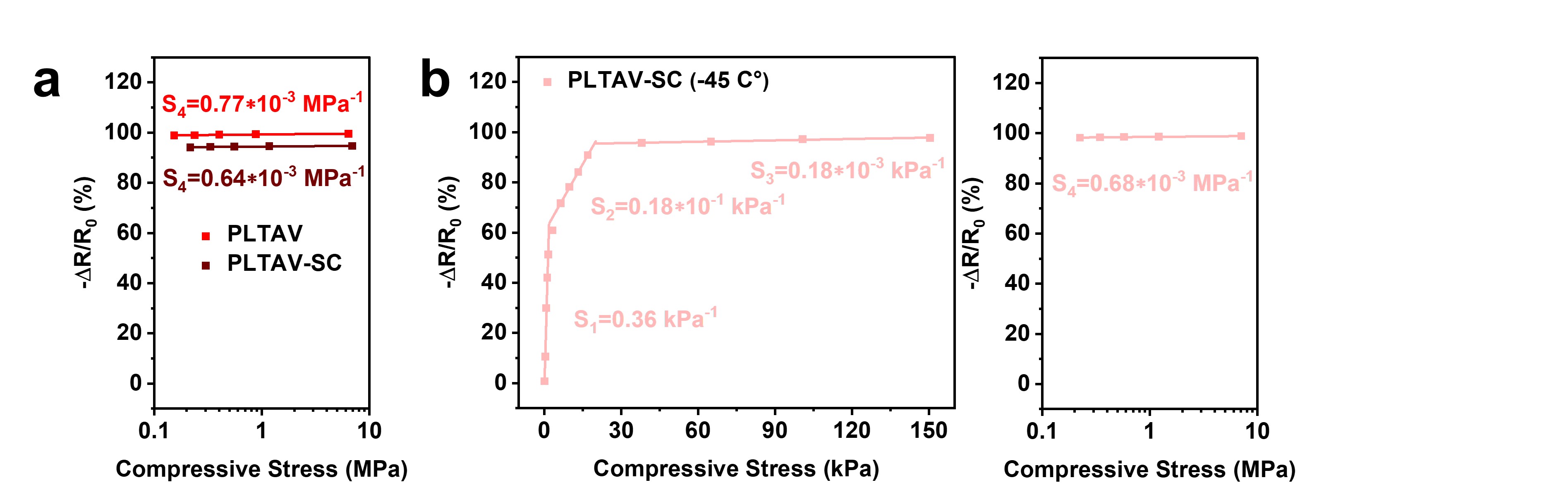


**Figure S36.** a) Relative resistance changes of PLTAV and PLTAV-SC sensors with compressive stress. b) Relative resistance variation of PLTAV-SC sensor with compressive stress at -45 ºC.

**Figure S37.** Variation of real-time relative resistance of PLTAV and PLTAV-SC sensors with wrist pulse beating.


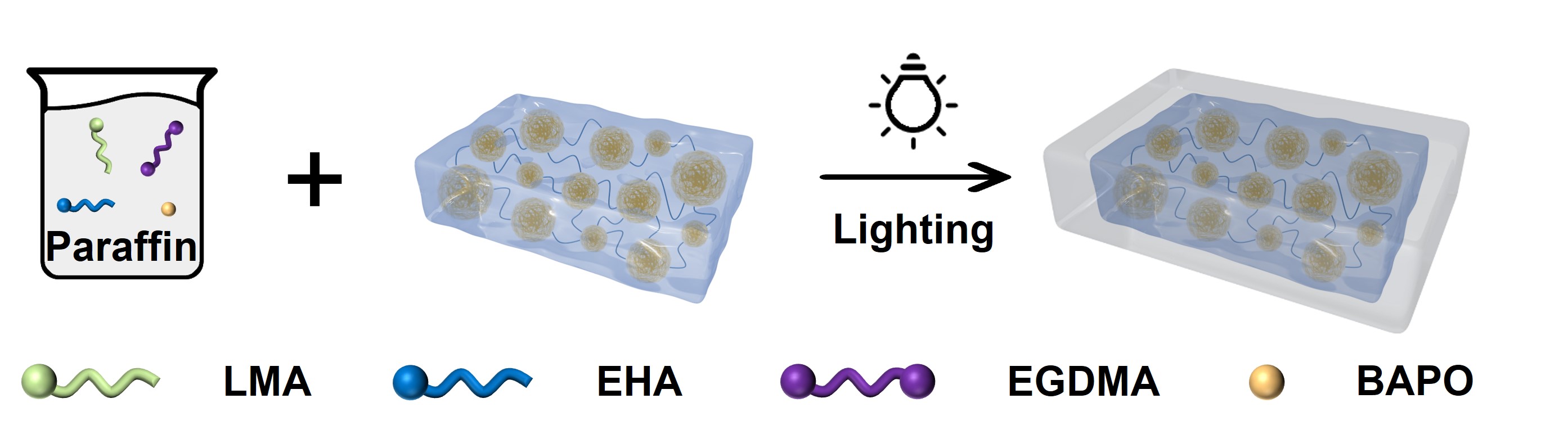


**Figure S38.** Preparation process of a hydrophobic coating.


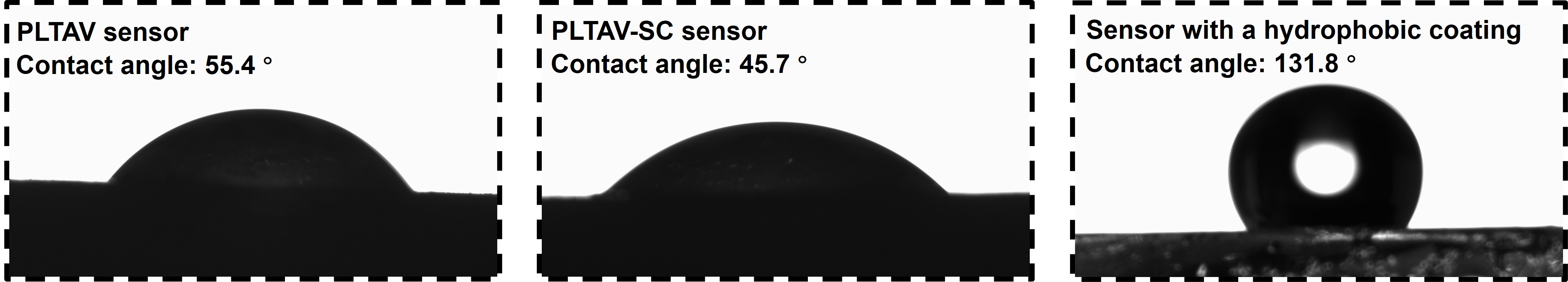


**Figure S39.** Static water contact angles of PLTAV and PLTAV-SC sensors and a sensor with a hydrophobic coating.


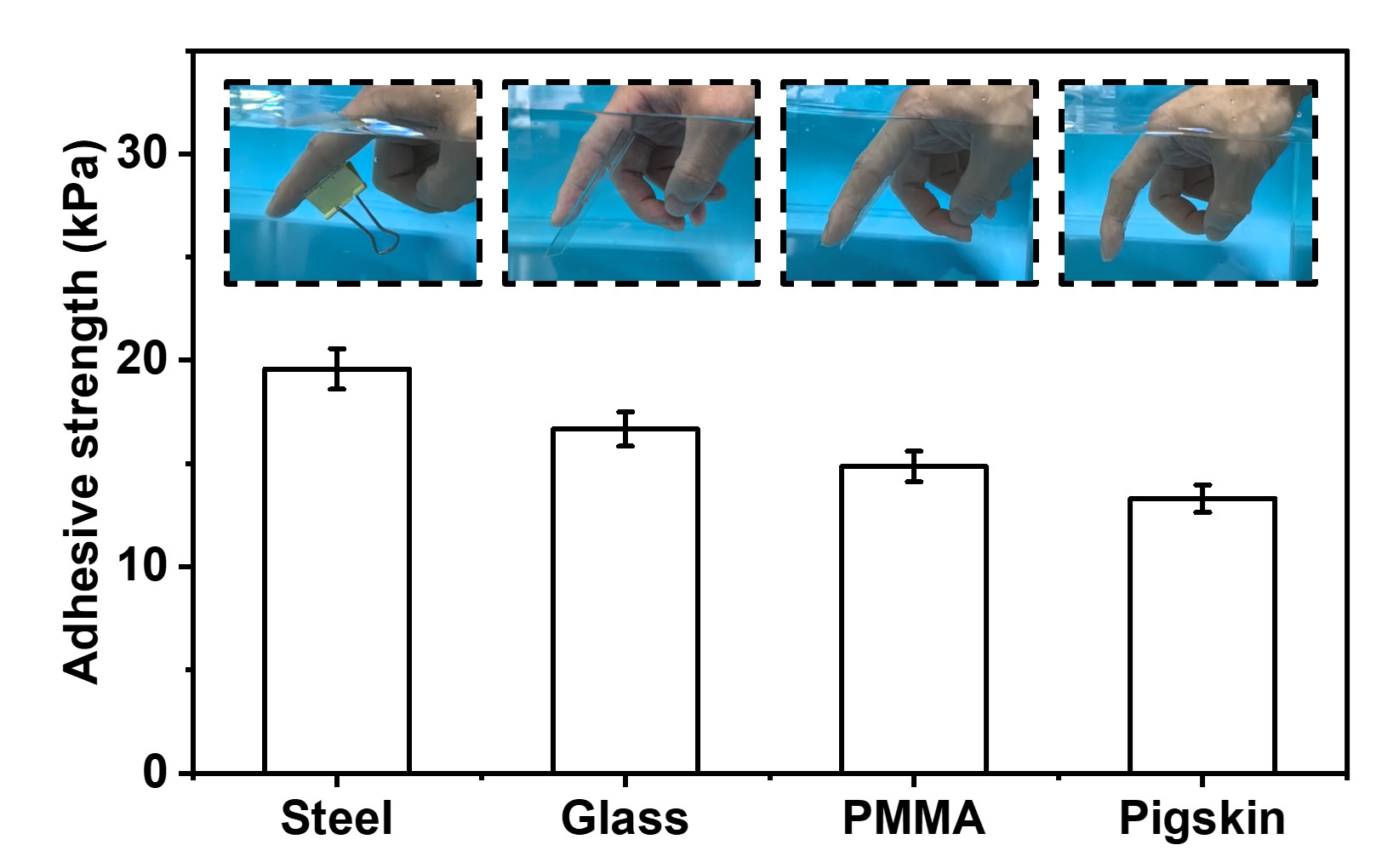


**Figure S40.** Underwater adhesion performance of a sensor with a hydrophobic coating.


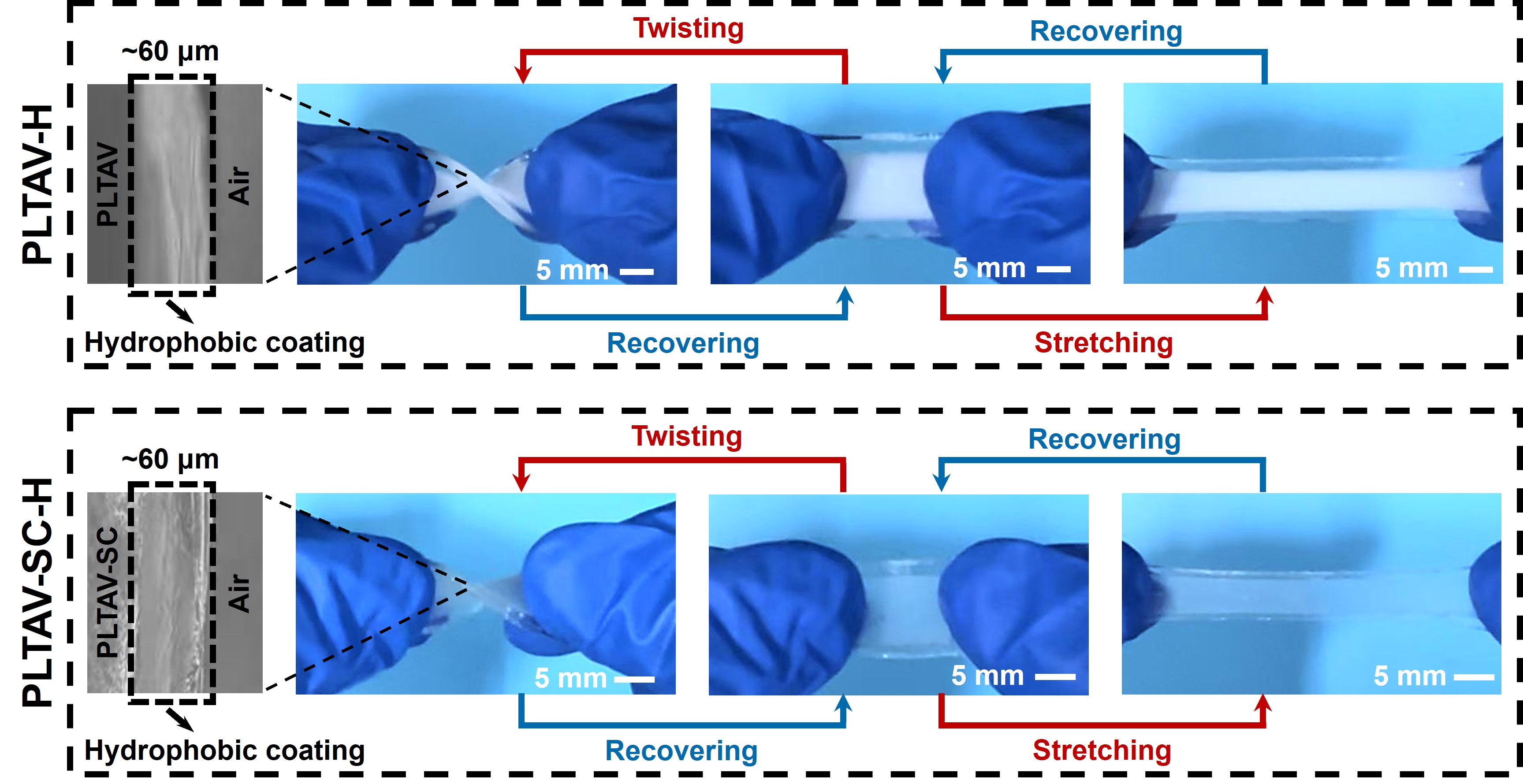


**Figure S41.** OM images, flexibility, and stretchability of PLTAV-H and PLTAV-SC-H sensors.


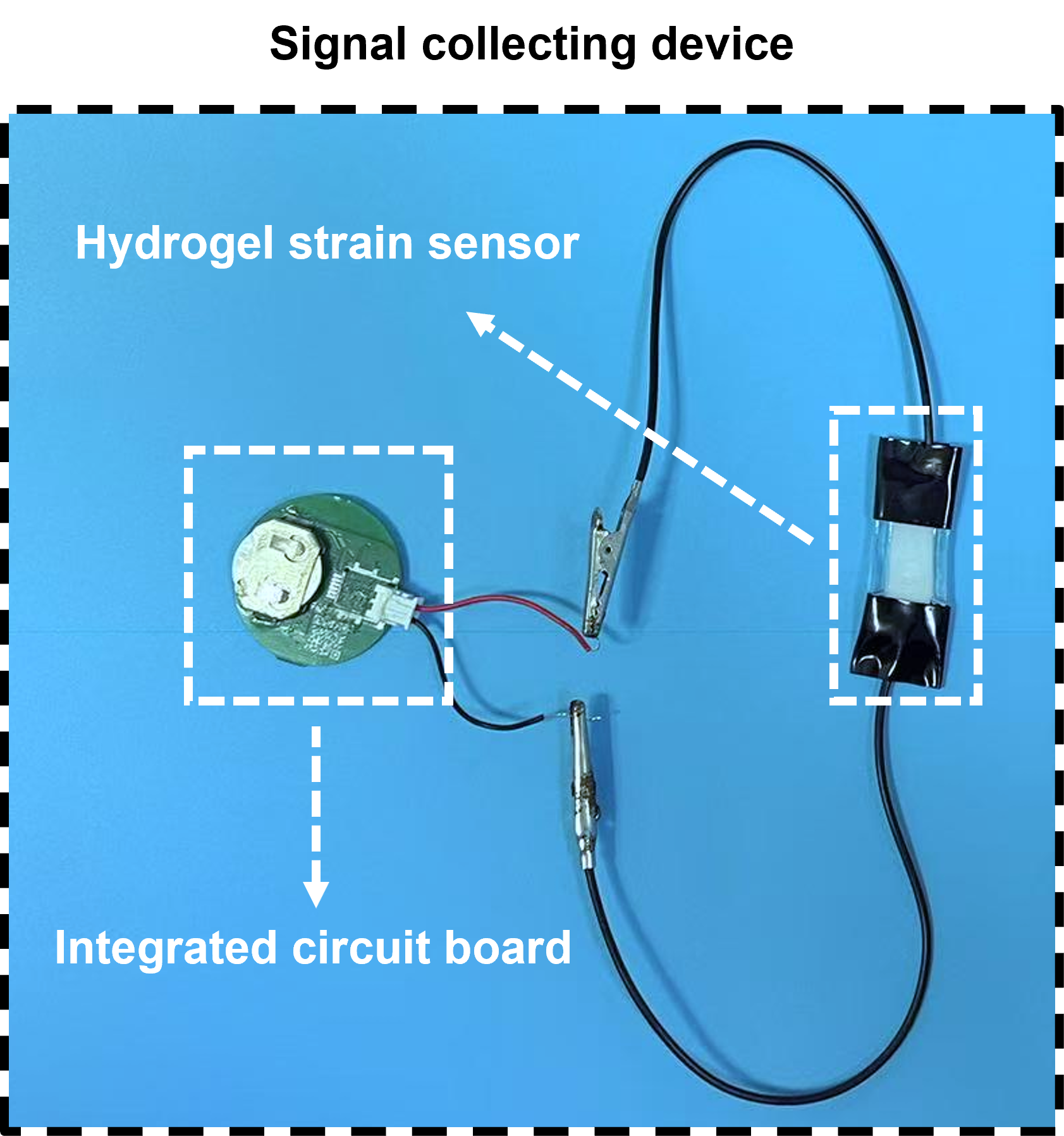


**Figure S42.** Signal collecting device in a wireless strain sensing system.

**Table S1.** Mechanical properties of PLTAV hydrogel with different volume ratios of hydrophobic precursor solution to hydrophilic precursor solution (V_Hydrophobic_:V_Hydrophilic_).

| V_Hydrophobic_:V_Hydrophilic_ | Tensile strength (MPa) | Tensile strain (%) | Toughness (MJ/m^3^) | Modulus (kPa) |
| --- | --- | --- | --- | --- |
| 0:1 | 0.050 | 1173 | 0.34 | 7.71 |
| 1:7 | 0.468 | 1220 | 2.4 | 91.24 |
| **1:6** | **0.465** | **1368** | **2.64** | **88.13** |
| 1:5 | 0.380 | 1518 | 2.32 | 66.62 |
| 1:4 | 0.187 | 1249 | 1.29 | 55.79 |

The tensile strength, tensile strain, toughness, and modulus of the PLTAV hydrogels with different volume ratios of hydrophobic precursor solution to hydrophilic precursor solution are presented in Table S1. The introduction of hydrophobic precursor solutions enhances the tensile strength, tensile strain, toughness, and modulus of the hydrogel. Increased values depend on the hydrophobic precursor solution content. Therefore, the as-prepared PLTAV hydrogel exhibits tunable mechanical properties. When the volume ratio of hydrophobic precursor solution to hydrophilic precursor solution reaches 1:6, the PLTAV hydrogel possesses the highest toughness, and its tensile strength, tensile strain, and modulus remain competitive. Thus, this ratio was selected to prepare the PLTAV hydrogel.

**Table S2.** Mechanical properties of PLTAV hydrogel with different types and dosages of crosslinking agent, initiator dosages, and solid contents of hydrophilic precursor solution.

| Crosslinker | | Initiator (wt% to monomer) | Solid content of hydrophilic precursor solution (wt%) | Tensile strength (MPa) | Tensile strain  (%) | Toughness (MJ/m^3^) | Modulus (kPa) |
| --- | --- | --- | --- | --- | --- | --- | --- |
| Type | Content (wt% to monomer) |  |  |  |  |  |  |
| EGDMA  (Hydrophobic) | 0.1 | 1 | 40 | 0.293 | 1277 | 1.71 | 53.48 |
| PEGDA  (Hydrophilic) | 0.1 | 1 | 40 | 0.403 | 677 | 1.27 | 148.38 |
| **PEGDA** | **0.05** | **1** | **40** | **0.465** | **1368** | **2.64** | **88.13** |
| PEGDA | 0 | 1 | 40 | 0.102 | 1412 | 0.75 | 29.71 |
| PEGDA | 0.05 | 0.5 | 40 | 0.119 | 1529 | 0.87 | 12.47 |
| PEGDA | 0.05 | 1.5 | 40 | 0.240 | 1335 | 1.40 | 54.24 |
| PEGDA | 0.05 | 1 | 20 | 0.063 | 1545 | 0.46 | 10.97 |
| PEGDA | 0.05 | 1 | 50 | - | - | - | - |

As shown in Table S2, compared to the hydrophobic EGDMA, the use of the hydrophilic PEGDA significantly enhances the tensile strength, toughness, and modulus of the PLTAV hydrogel while slightly reducing the tensile strain of the hydrogel. Excessive PEGDA renders the PLTAV hydrogel rigid, adversely affecting its mechanical properties. Additionally, an unsuitable initiator dosage can result in weakened mechanical properties. The higher the solid content of the hydrophilic precursor solution, the more the chain entanglements, and the better the mechanical properties.^[2-4]^ However, an excessive solid content in the hydrophilic precursor solution leads to the formation of the unstable emulsion. Therefore, in this work, the PEGDA was chosen to prepare the PLTAV hydrogel. The dosages of the PEGDA and initiator were set at 0.05 wt% and 1 wt%, respectively, based on the monomer mass. The solid content of the hydrophilic precursor solution was set at 40 wt%.

**Table S3.** Comparison of water content, toughness, hysteresis, tensile strain, and compressive strain of PLTAV and PTLAV-SC hydrogels with previously reported low-hysteresis hydrogels, organogels, and ionogels.^[3-19]^

| Gel type | Sample name | Toughness (MJ/m^3^) | Hysteresis (%) | Tensile strain (%) | Compressive strain (%) | Water content (wt%) | Reference |
| --- | --- | --- | --- | --- | --- | --- | --- |
| Hydrogel | **PLTAV** | **2.64** | **4.7** | **1368** | **99.9** | **90.4** | **This work** |
|  | **PLTAV-SC** | **6.10** | **6.6** | **2021** | **99.9** | **20.0** | **This work** |
|  | APC27 | ~0.30 | 1.0 | 440 | 92.0 | 94.0 | [3] |
|  | HEDN | 2.49 | 1.0 | 325 | 83.0 | 90.0 | [4] |
|  | P(AAm-THMA-ILs)/PP | 1.22 | 5.0 | 1015 | 97.1 | 71.0 | [5] |
|  | PAM-TSASN-LiCl | ~0.01 | 8.0 | 1200 | 80.0 | 85.9 | [6] |
|  | PATV | 0.45 | 9.0 | 900 | 80.0 | 81.7 | [7] |
|  | LSN-Fe/PAM | ~0.08 | 15 | 1100 | 80.0 | 73.2 | [8] |
|  | 3A/ADSP/LiCl | ~0.01 | 2.2 | 770 | 80.0 | 81.5 | [9] |
|  | APC23 | 2.50 | 6.0 | 1250 | 97.0 | ~62.0 | [10] |
|  | PM | 1.34 | 7.0 | 900 | - | ~50.0 | [11] |
|  | CPCa | 4.67 | 5.4 | ~900 | - | ~20.8 | [12] |
| Organogel | OH-PLT | ~0.16 | ~10.0 | 560 | 80.0 | ~37.0 | [13] |
|  | PHEA-TA@MXene | ~0.28 | ~5.0 | 690 | 60.0 | - | [14] |
|  | Organo-hydrogel | 0.48 | ~4.0 | ~800 | 90.0 | ~64.6 (Gly/water) | [15] |
|  | P(AAm-co-AAc)/PANI | 1.58 | ~19.7 | ~300 | 90.0 | ~42.6  (Gly/water) | [16] |
| Ionogel | PAU | ~1.30 | 7.9 | ~550 | - | ~71.6 (ionic liquid) | [17] |
|  | EA-PR-IL | ~0.09 | 7.0 | 550 | - | ~48.8 (ionic liquid) | [18] |
|  | BHIG | ~0.93 | ~15.0 | 578 | ~37.0 | ~50.0 (ionic liquid) | [19] |

**Table S4.** The comparison of design concept, morphology, and stress-absorbing component of PLTAV hydrogel with previously reported low-hysteresis hydrogels.^[3-8,10-12,20-22]^

| Structural design | Special morphology | Stress-absorbing component | Reference |
| --- | --- | --- | --- |
| **Epithelium-like** | **Cell-like particle** | **Cell-like particle** | **This work** |
| Double-network structure (hydrophilic networks) | No | One of the networks | [4,7] |
| Double-network structure (hydrophilic and hydrophobic networks) | Hydrophobic domain | Hydrophobic network | [20,21] |
| Nanocomposite structure | Nanoparticle | Nanoparticle | [3,5,6,8,10,11] |
| Slide-ring structure | No | Slide-ring | [22] |
| Phase-separation structure | Microphase | Microphase | [12] |

**Table S5.** Comparison of linear strain-range, response time, recovery time, self-powered strain sensing, visual strain sensing, and anti-freezing abilities of PLTAV and PLTAV-SC strain sensors with previously reported strain sensors.^[3,5-7,23-29]^

| Sensor name | Linear strain range (%) | Response time (ms) | Recovery time (ms) | Self-powered strain sensing | Visual strain sensing | Anti-freezing | Reference |
| --- | --- | --- | --- | --- | --- | --- | --- |
| **PLTAV** | **0.5-1300** | **38** | **40** | **Yes** | **Yes** | **No** | **This work** |
| **PLTAV-SC** | **0.7-1500** | **19** | **21** | **Yes** | **Yes** | **Yes** | **This work** |
| APC27 | 0-100 | 340 | 310 | No | No | No | [3] |
| P(AAm-THMA-ILs)/PP | 0-200 | 65 | 40 | Yes | No | No | [5] |
| PAM-TSASN-LiCl | 0-200 | 210 | 210 | No | No | No | [6] |
| PATV | 0-200 | 180 | 190 | Yes | No | No | [7] |
| PVA/PSBMA-H_2_SO_4_ | 0-150 | 160 | 220 | No | No | No | [23] |
| PAM-HA-Zn | 0-100 | ~382 | ~488 | No | No | No | [24] |
| PAATC | 0-400 | 450 | 350 | Yes | No | No | [25] |
| STSMH | 0-50 | 169 | 250 | No | Yes | No | [26] |
| PSZC | 0-500 | 140 | 130 | No | Yes | Yes | [27] |
| P(AA-co-Am)/Ca^2+^ | 0-150 | ~89 | ~97 | No | No | Yes | [28] |
| PR-Gel | 0-200 | - | - | No | No | Yes | [29] |

**References**

[1] Z. Wang, H. Zhou, D. Liu, X. Chen, D. Wang, S. Dai, F. Chen, B. B. Xu, *Adv. Funct. Mater.* **2022**, *32*, 2201396.

[2] J. Kim, G. Zhang, M. Shi, Z. Suo, *Science* **2021**, *374*, 212.

[3] P. Liu, Y. Zhang, Y. Guan, Y. Zhang, *Adv. Mater.* **2023**, *35*, 2210021.

[4] R. Zhu, D. Zhu, Z. Zheng, X. Wang, *Nat. Commun.* **2024**, *15*, 1344.

[5] W. Wang, P. Guo, X. Liu, M. Chen, J. Li, Z. Hu, G. Li, Q. Chang, K. Shi, X. Wang, K. Lei, *Adv. Funct. Mater.* **2024**, *34*, 2316346.

[6] S. Han, H. Tan, J. Wei, H. Yuan, S. Li, P. Yang, H. Mi, C. Liu, C. Shen, *Adv. Sci.* **2023**, *10*, 2301713.

[7] S. Han, Y. Hu, J. Wei, S. Li, P. Yang, H. Mi, C. Liu, C. Shen, *Adv. Funct. Mater.* **2024**, *34*, 2401607.

[8] H. Zhao, S. Hao, Q. Fu, X. Zhang, L. Meng, F. Xu, J. Yang, *Chem. Mater.* **2022**, *34*, 5258.

[9] Y. Wang, L. Song, Q. Wang, L. Wang, S. Li, H. Du, C. Wang, Y. Wang, P. Xue, W. Nie, X. Wang, S. Tang, *Carbohydr. Polym.* **2023**, *318*, 121106.

[10] R. Liu, H. Wang, W. Lu, L. Cui, S. Wang, Y. Wang, Q. Chen, Y. Guan, Y. Zhang, *Chem. Eng. J.* **2021**, *415*, 128839.

[11] J. Zou, X. Jing, S. Li, P. Feng, Y. Chen, Y. Liu, *Small* **2024**, *20*, 2401622.

[12] Q. Wu, Y. Xu, S. Han, A. Chen, J. Zhang, Y. Chen, X. Yang, L. Guan, *ACS Nano* **2024**, *18*, 31148.

[13] Y. Zhao, Q. Zhao, S. Peng, H. Zhou, L. Yang, *J. Mater. Chem. C* **2023**, *11*, 16135.

[14] Y. Liu, G. Tian, Y. Du, P. Shi, N. Li, Y. Li, Z. Qin, T. Jiao, X. He, *Adv. Funct. Mater.* **2024**, *34*, 2315813.

[15] S. Li, Z. Xiao, H. Yang, C. Zhu, G. Chen, J. Zheng, J. Ren, W. Wang, Y. Cong, L. Ali Shah, J. Fu, *Chem. Eng. J.* **2024**, *499*, 156581.

[16] G. Chen, Y. Zhang, S. Li, J. Zheng, H. Yang, J. Ren, C. Zhu, Y. Zhou, Y. Chen, J. Fu, *Adv. Mater.* **2024**, *36*, 2408193.

[17] B. Sun, K. Liu, B. Wu, S. Sun, P. Wu, *Adv. Mater.* **2024**, *36*, 2408826.

[18] R. Du, T. Bao, T. Zhu, J. Zhang, X. Huang, Q. Jin, M. Xin, L. Pan, Q. Zhang, X. Jia, *Adv. Funct. Mater.* **2023**, *33*, 2212888.

[19] C. Zhou, X. Song, R. Wei, S. Liu, Z. Wu, H. Chen, *Chem. Eng. J.* **2024**, *499*, 155992.

[20] X. Hou, B. Huang, L. Zhou, S. Liu, J. Kong, C. He, *Adv. Mater.* **2023**, *35*, 2301532.

[21] G. Zhang, J. Steck, J. Kim, C. H. Ahn, Z. Suo, *Sci. Adv.* **2023**, *9*, eadh7742.

[22] C. Liu, N. Morimoto, L. Jiang, S. Kawahara, T. Noritomi, H. Yokoyama, K. Mayumi, K. Ito, *Science* **2021**, *372*, 1078.

[23] J. Ren, G. Chen, H. Yang, J. Zheng, S. Li, C. Zhu, H. Yang, J. Fu, *Adv. Mater.* **2024**, *36*, 2412162.

[24] S. Guan, C. Xu, X. Dong, M. Qi, *J. Mater. Chem. A* **2023**, *11*, 15404.

[25] H. Yuan, S. Han, J. Wei, S. Li, P. Yang, H. Mi, C. Liu, C. Shen, *J. Energy Chem.* **2024**, *94*, 136.

[26] L. Bai, Y. Jin, X. Shang, H. Jin, L. Shi, Y. Li, Y. Zhou, *Nano Energy* **2022**, *104*, 107962.

[27] Y. Zhang, X. Jing, J. Zou, P. Feng, G. Wang, J. Zeng, L. Lin, Y. Liu, H. Mi, S. Nie, *Adv. Funct. Mater.* **2024**, *34*, 2410698.

[28] L. Zhou, B. Zhao, J. Liang, F. Lu, W. Yang, J. Xu, J. Zheng, Y. Liu, R. Wang, Z. Liu, *Mater. Horiz.* **2024**, *11*, 3856.

[29] X. Xiong, Y. Chen, Z. Wang, H. Liu, M. Le, C. Lin, G. Wu, L. Wang, X. Shi, Y. Jia, Y. Zhao, *Nat. Commun.* **2023**, *14*, 1331.
